# Targeting YAP1‐regulated Glycolysis in Fibroblast‐Like Synoviocytes Impairs Macrophage Infiltration to Ameliorate Diabetic Osteoarthritis Progression

**DOI:** 10.1002/advs.202304617

**Published:** 2023-12-03

**Authors:** Jie Yang, Shanshan Li, Zhenyan Li, Lutian Yao, Meijing Liu, Kui‐Leung Tong, Qiutong Xu, Bo Yu, Rui Peng, Tao Gui, Wang Tang, Yidi Xu, Jiaxu Chen, Jun He, Kewei Zhao, Xiaogang Wang, Xiaoying Wang, Zhengang Zha, Huan‐Tian Zhang

**Affiliations:** ^1^ Department of Bone and Joint Surgery the First Affiliated Hospital of Jinan University Key Laboratory of Regenerative Medicine of Ministry of Education Jinan University Guangzhou Guangdong 510630 China; ^2^ State Key Laboratory of Pulp and Paper Engineering South China University of Technology Guangzhou 510640 China; ^3^ Department of Orthopedics The First Hospital of China Medical University Shenyang 110001 China; ^4^ Key Laboratory of Big Data‐Based Precision Medicine School of Engineering Medicine Beihang University Beijing 100191 China; ^5^ Clinical Research Platform for Interdisciplinary of Stomatology the First Affiliated Hospital of Jinan University and Department of Stomatology Jinan University Guangzhou 510632 China; ^6^ Guangzhou Key Laboratory of Formula‐Pattern Research Center School of Traditional Chinese Medicine Jinan University Guangzhou 510640 China; ^7^ Institute of Laboratory Animal Science Jinan University Guangzhou 510632 China; ^8^ Guangzhou Key Laboratory of Chinese Medicine Research on Prevention and Treatment of Osteoporosis the Third Affiliated Hospital of Guangzhou University of Chinese Medicine Guangzhou 510375 China

**Keywords:** diabetic osteoarthritis, fibroblast‐like synoviocytes, glycolysis, macrophages infiltration, YAP1

## Abstract

The interplay between immune cells/macrophages and fibroblast‐like synoviocytes (FLSs) plays a pivotal role in initiating synovitis; however, their involvement in metabolic disorders, including diabetic osteoarthritis (DOA), is largely unknown. In this study, single‐cell RNA sequencing (scRNA‐seq) is employed to investigate the synovial cell composition of DOA. A significant enrichment of activated macrophages within eight distinct synovial cell clusters is found in DOA synovium. Moreover, it is demonstrated that increased glycolysis in FLSs is a key driver for DOA patients’ synovial macrophage infiltration and polarization. In addition, the yes‐associated protein 1 (YAP1)/thioredoxin‐interacting protein (TXNIP) signaling axis is demonstrated to play a crucial role in regulating glucose transporter 1 (GLUT1)‐dependent glycolysis in FLSs, thereby controlling the expression of a series of adhesion molecules such as intercellular adhesion molecule‐1 (ICAM‐1) which may subsequently fine‐tune the infiltration of M1‐polarized synovial macrophages in DOA patients and *db/db* diabetic OA mice. For treatment, M1 macrophage membrane‐camouflaged Verteporfin (Vt)‐loaded PLGA nanoparticles (MVPs) are developed to ameliorate DOA progression by regulating the YAP1/TXNIP signaling axis, thus suppressing the synovial glycolysis and the infiltration of M1‐polarized macrophages. The results provide several novel insights into the pathogenesis of DOA and offer a promising treatment approach for DOA.

## Graphical abstract

Enrichment of M1 macrophage and enhanced glycolysis of fibroblast‐like synoviocytes (FLSs) play key roles in pathogenesis of diabetic osteoarthritis (DOA). Mechanistically, yes‐associated protein 1 (YAP1)/thioredoxin‐interacting protein (TXNIP) signaling axis regulates the glycolysis of FLSs thus fine‐tuning the M1 macrophage recruitment and infiltration. Furthermore, M1 macrophage membrane‐camouflaged Vt‐loaded nanoparticles could significantly alleviate the DOA by suppressing the YAP1/TXNIP signaling regulated synovial glycolysis and M1 macrophages infiltration.

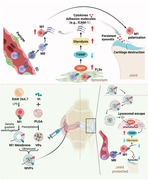

## 1 Introduction

Osteoarthritis (OA) is the most common painful and debilitating disease among elderly individuals, and it is now considered a whole‐joint disease characterized by articular cartilage loss, subchondral bone dysfunction, and synovial inflammation.^[^ ^1^ ^]^ In recent years, studies have indicated that metabolic diseases, particularly type 2 diabetes mellitus (T2DM), which is widely recognized as a chronic low‐grade inflammatory status, serve as independent risk factors for the pathogenesis of OA.^[^ ^2^ ^]^ In addition, the interplay between joint degeneration and metabolic disturbances in T2DM can aggravate cartilage destruction and synovial inflammation, leading to the novel concept of diabetic OA (DOA).^[^ ^3^ ^]^ Obesity, often associated with T2DM, can exacerbate DOA due to increased mechanical stress on the joints.^[^ ^4^ ^]^ Additionally, diabetes can stimulate the generation of proinflammatory factors such as cytokines, adipokines, fatty acids, and reactive oxygen species, which can lead to persistent synovitis and further degeneration of the cartilage.^[^ ^5^ ^]^ Studies have highlighted the important role of both resident synovial and peripheral macrophages in fine‐tuning the synovial environment and preserving joint homeostasis throughout the onset and advancement of OA and DOA,^[^ ^6^ ^]^ but the precise mechanisms remain unclear.

The emergence of single‐cell RNA sequencing (scRNA‐seq) technology has enabled researchers to identify and characterize diverse cell populations within the synovium, including fibroblast‐like synoviocytes (FLSs), endothelial cells (ECs), pericytes, macrophages, dendritic cells, mast cells, natural killer cells, T cells, and B cells.^[^ ^7^ ^]^ Cell–cell interactions and/or cross‐talk between different cell populations in the synovium are pivotal for triggering synovitis (synovial hyperplasia).^[^ ^8^ ^]^ For instance, tissue‐resident FLSs not only constitute the architecture of the synovium but also play a crucial functional role in regulating the metabolism of macrophages. Conversely, inflammatory macrophages can modulate the proliferation and behavior of synovial FLSs.^[^ ^9^ ^]^ Moreover, their interaction creates a cytokine network that activates an inflammatory response during synovitis.^[^ ^10^ ^]^ Metabolically, a transition from oxidative phosphorylation to glucose transporter (GLUT)‐mediated glycolysis has been observed in synovium during synovitis. This metabolic shift allows for the generation of more energy and intermediates, which are necessary for the biosynthesis of inflammatory mediators that contribute to persistent synovitis.^[^ ^11^ ^]^

Two major subsets of macrophages known as M1 (pro‐inflammatory/classically activated) and M2 (anti‐inflammatory/alternatively activated), play pivotal roles in regulating the synovial microenvironment and joint hemostasis.^[^ ^12^ ^]^ Increasing evidence has suggested that the accumulation of M1 macrophages is an essential prerequisite for amplifying the inflammatory response and consequently results in chronic synovitis.^[^ ^8a^ ^]^ In addition, the M1/M2 ratio is significantly elevated in synovial fluid and peripheral blood of OA patients compared to healthy controls, which positively correlates with OA severity.^[^ ^13^ ^]^ On the other hand, metabolic diseases such as T2DM can facilitate macrophage infiltration into joint synovium and drive OA onset and progression.^[^ ^14^ ^]^ In T2DM, elevated levels of cell surface adhesion molecules such as intercellular adhesion molecule‐1 (ICAM‐1) and vascular cell adhesion molecule‐1 (VCAM‐1) in circulation can facilitate macrophages recruitment into joints, thus propagating the inflammatory response.^[^ ^15^ ^]^ We have recently demonstrated the essential role of ICAM‐1 in triggering the vicious cycle of inflammation and driving cartilage degradation in DOA.^[^ ^16^ ^]^ However, the precise contributions of macrophages to DOA and their potential as therapeutic targets remain incompletely understood.

As one of the most important mechanotransduction molecules in OA, yes‐associated protein 1 (YAP1) has been demonstrated to be essential for regulating mechanical loading, synovial metabolism, inflammation, and macrophage polarization.^[^ ^17^ ^]^ YAP1 stimulates glucose metabolism by enhancing glucose uptake and glycolysis, potentially through the regulation of GLUTs and key glycolysis enzymes in various inflammatory diseases.^[^ ^18^ ^]^ Therefore, targeting YAP1 is a promising approach for alleviating the corresponding diseases, including metabolic OA. Recently, macrophage‐membrane‐coated nanoparticles have gained attention as potential therapeutic tools for inflammatory diseases, including OA.^[^ ^19^ ^]^ These nanoparticles inherit biological functionalities like cell‐to‐cell communication from source cells and have superior biocompatibility, prolonged circulation, and targeted delivery abilities.^[^ ^20^ ^]^ These outstanding properties make them promising tools for the treatment of OA.

**Scheme 1:**
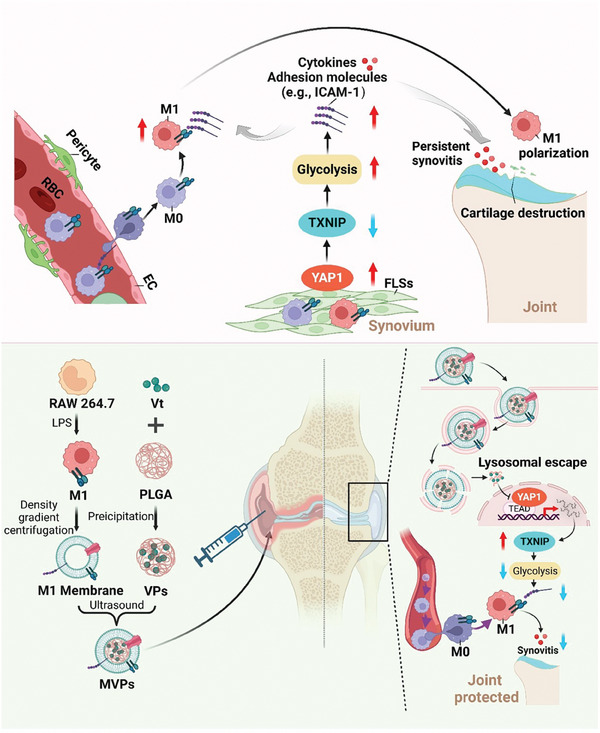
A schematic diagram of targeting YAP1‐regulated glycolysis in FLSs impairs macrophage infiltration and polarization to ameliorate DOA progression.

The present study utilized scRNA‐seq to investigate the cellular dynamics in the synovium of DOA. The results revealed increased pro‐inflammatory M1 macrophages and enhanced glycolysis of FLSs, indicating their involvement in DOA pathogenesis. In addition, YAP1/thioredoxin‐interacting protein (TXNIP) signaling was demonstrated to be required for the regulation of FLSs glycolysis, which in turn influences M1 macrophage infiltration and polarization. Further, we developed an **M** 1 macrophage membrane‐camouflaged **V** erteporfin (Vt)‐loaded **P** LGA nanoparticle **s** (MVPs) to ameliorate DOA progression by regulating the YAP1/TXNIP signaling axis, consequently suppressing synovial glycolysis and the infiltration of M1‐polarized macrophages (**Scheme** **1**). Collectively, our results provide novel insights into the mechanisms underlying DOA pathogenesis and offer a promising therapeutic strategy for DOA treatment.

## 2 Results

### 2.1 scRNA‐Seq Reveals M1‐Macrophage Enrichment in the Synovium of DOA

**Figure 1:**
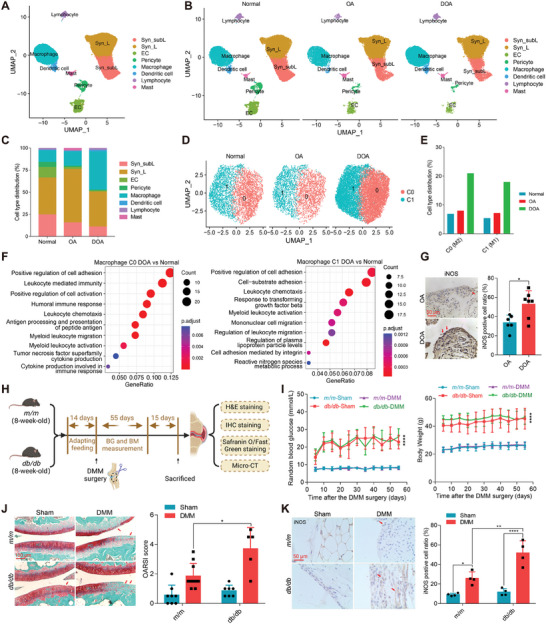
M1 macrophages are enriched in the synovium of DOA. A) Uniform Manifold Approximation and Projection (UMAP) identifies eight cell clusters in the synovium of normal, OA, and DOA patients. Each cluster is represented by a different color, and the general identity of each cell cluster is shown on the right. B) UMAP visualization of the cell types identified in the synovium of normal (*n* = 3), OA (*n* = 2), and DOA patients (*n* = 3). The general identity of each cell cluster is provided on the right. C) The proportion of each cell type in the synovium across patients is shown. D) UMAP visualization of the macrophages, which are divided into two cell clusters labeled C0 and C1. E) The percentage of C0 and C1 macrophages among the total cell population in the synovium is presented for normal, OA, and DOA patients. F) Gene ontology enrichment of differentially expressed genes in C0 and C1 macrophages isolated from the synovium of normal, OA, and DOA patients. G) Representative images of IHC staining and the quantification of CD86^+^ and iNOS^+^ cells in the synovium of OA and DOA patients (*n* ≥ 5). H) A schematic diagram illustrating the experimental timelines of an OA model induced by destabilization of the medial meniscus (DMM) in the diabetic *(db/db)* mice (BKS‐Lepr em2Cd479/Nju, T002407) and non‐diabetic (*m*/*m)* mice (C57BLKS/JNju, N000214). I) Blood glucose levels and body weight of mice were measured for 55 days after the DMM surgery (*n* > 6). J) Safranin O/Fast Green staining and scoring of OARSI grade in mice subjected to sham operation or DMM surgery, with or without diabetes mellitus (*n* ≥ 5). K) Representative images of iNOS immunostaining and quantification of positive cells in knee joints of the indicated mouse groups (*n* = 4). Data are presented as mean ± SD, and dots represent individual mice or human samples. *p < 0.05, **p < 0.01, and ****p < 0.0001. Student's t‐test for (G); two‐way ANOVA for (I–K).

DOA patients commonly experience worsened synovitis, exacerbated cartilage loss, and a higher surgical rate,^[^ ^5a^ ^]^ but the underlying mechanisms are largely unknown. In an aged‐matched cohort, we were able to confirm that the synovitis score was higher in DOA patients than in OA patients as determined by HE staining (Table S1 and Figure S1A, Supporting Information). Next, to investigate the mechanism, scRNA‐seq was conducted using synovial specimens obtained from patients with various conditions, including those with meniscus or anterior cruciate ligament (ACL) injury (3 cases, 2–4 weeks post‐injury, representing normal synovium), individuals with OA (2 cases) and DOA (3 cases) who underwent either arthroscopy or total knee arthroplasty (OA and DOA) surgery (Table S2 and Figure S1B,C, Supporting Information). By combining the dataset from normal, OA, and DOA groups, we were able to identify eight distinct cell clusters, including synovial sublining (Syn_subL) FLSs, synovial lining (Syn_L) FLSs, ECs, pericytes, macrophages, dendritic cells, lymphocytes, and mast cells, totaling of 83 097 cells (**Figure** **1**A; Figure S2, Supporting Information). Notably, the synovium of DOA patients exhibited a significantly higher percentage of macrophages (44.9%) compared to OA patients (16.7%) and individuals with normal synovium (13.9%) (Figure 1B,C; Figure S2, Supporting Information). Previously, little was known about the changes in immune cells during the pathogenesis of DOA.^[^ ^21^ ^]^ Herein, the use of scRNA‐seq provided a more comprehensive understanding of the increased presence of macrophages within the DOA synovium (Figure 1C). Next, we were interested in exploring the specific subpopulations of macrophages that might contribute to the observed inflammatory response in DOA. Macrophages in DOA exhibited higher scores of Macrophage Polarization Index (MPI) and Activation‐induced Macrophage Differentiation Index (AMDI), as defined by MacSpectrum,^[^ ^22^ ^]^ indicating a greater degree of inflammatory polarization and more advanced differentiation status of macrophages in DOA condition (Figure S3A, Supporting Information). Notably, the macrophages could be further categorized into two subclusters: C0 and C1. The relative proportion of C0 and C1 was dramatically elevated in the synovium of patients with DOA (21.0% and 18.0%) compared to those with normal synovium (6.9% and 5.4%) (Figure 1D,E). Gene ontology (GO) term analysis revealed that C0 was a type of phagocytic macrophage with functions related to phagocytosis and humoral immune response; whereas C1 was an inflammatory macrophage primarily involved in cytokine production and the inflammatory response (Figure S3B–D, Supporting Information). In addition, gene set variation analysis (GSVA) and gene set enrichment analysis (GSEA) further indicated that, compared to macrophages in normal synovium, DOA macrophages exhibited an enrichment of signaling pathways associated with cell adhesion, activation of immune cells, and cytokines production were enriched in the (Figure 1F; Figure S3E–G, Supporting Information). Given the upregulation of *IL‐1β*, *IL‐10*, *MMP1*, *MMP3*, *MMP9*, and *MMP13* expression in DOA synovium (Figure S4, Supporting Information), it is reasonable to assume that the above signaling pathways might drive the onset of synovitis by increasing the secretion of inflammatory cytokines in the osteoarthritic joints.^[^ ^23^ ^]^

Furthermore, immunohistochemistry (IHC) staining was performed to validate the enrichment of activated macrophages using the typical markers of M1 polarized macrophages, such as a cluster of differentiation 86 (CD86) and inducible nitric oxide synthase (iNOS).^[^ ^12^, ^24^ ^]^ The CD86 and iNOS staining validated a higher abundance of M1 macrophages in DOA synovium compared to OA (Figure 1G; Figure S5A, Supporting Information). To further confirm this finding, we established a DOA mouse model using 10‐week‐old *m*/*m* control or *db*/*db* diabetic mice combined with the destabilization of the medial meniscus (DMM) surgery, followed by a series of histological evaluation at the endpoint (Figure 1H). Consistent with previous reports,^[^ ^25^ ^]^ *db*/*db* mice exhibited elevated blood glucose levels and body weight (Figure 1I). Moreover, histological staining revealed more severe synovial hyperplasia and substantial cartilage loss in the *db*/*db* mice in response to DMM induction, and lower bone density compared to non‐diabetic *m*/*m* mice, as determined by micro‐computed tomography (micro‐CT) analysis (Figure 1J; Figure S5B,C, Supporting Information). Accordingly, IHC staining and quantitative analysis suggested a marked increase in CD86‐ and iNOS‐positive cells (Figure 1K; Figure S5D, Supporting Information). These findings suggest that there is an enrichment in M1 macrophages in the DOA synovium, which may account for the exacerbated synovitis and the manifestation of the DOA phenotype.

### 2.2 Enhanced TXNIP/GLUT1‐Dependent Glycolysis in FLSs favors the Recruitment and Infiltration of Macrophages

**Figure 2:**
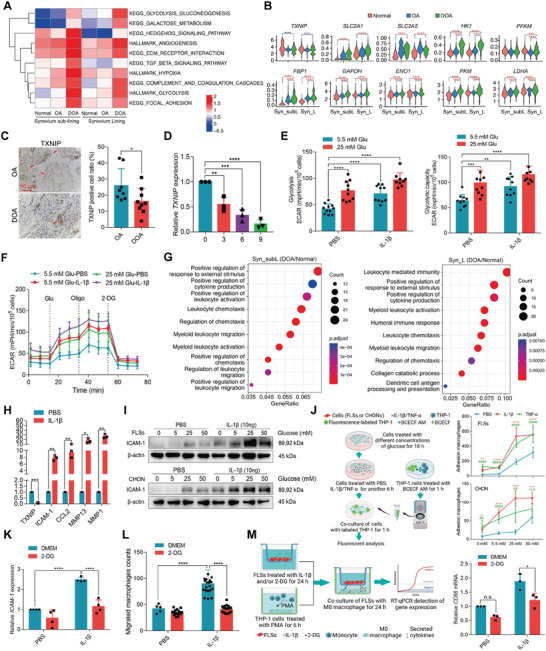
Enhanced TXNIP/GLUT1‐dependent glycolysis in FLSs facilitates the recruitment and infiltration of activated macrophages. A) GSVA analysis of synovial sublining and lining fibroblasts of patients with DOA to those of normal and OA patients. Red and blue colors indicate increased or decreased signaling pathways, respectively. B) Violin plots of the expression levels of the glycolytic gene (*TXNIP*, *SLC2A1/GLUT1*, *SLC2A5*, *HK1*, *PFKM, FPB1*, *GAPDH*, *ENO1*, *PKM*, and *LDHA*) in synovial sublining and lining fibroblasts of DOA compared to normal and OA. C) Representative images of IHC staining and quantification of TXNIP^+^ cells in the synovium of OA and DOA patients (*n* ≥ 8). D) *TXNIP* mRNA expression in the FLSs treated with IL‐1β for different durations (0, 3, 6, and 9 h) (*n* = 3). E,F) Seahorse analysis of extracellular acidification rate (ECAR), glycolysis, and glycolytic capacity in the FLSs treated with different glucose concentrations (5.5 or 25 mm) along with 10 ng mL^−1^ IL‐1β or PBS for 24 h. G) Gene ontology enrichment analysis on the differentially expressed genes in synovial sublining and lining fibroblasts of normal, OA, and DOA patients. H) *TXNIP*, *ICAM‐1*, *CCL2*, *MMP13*, and *MMP1* mRNA expression in the FLSs treated with IL‐1β for 24 h (*n* = 3). I) Immunoblotting of ICAM‐1 expression in FLSs and CHON after treatment with different glucose concentrations (0, 5.5, 25, and 50 mm) along with PBS or IL‐1β for 24 h. J) Schematic diagram of the monocyte adhesion assay is shown on the left panel. Adhesion macrophages were analyzed as in the right panel. * is the statistical analysis between IL‐1β and PBS, and # is the statistical analysis between TNF‐α and PBS. K) The *ICAM‐1* mRNA expression in the FLSs treated with DMEM or 2‐DG and IL‐1β or PBS for 24 h. L) The number of migrated macrophages was determined after treatment with 20 mm 2‐DG in the presence of IL‐1β stimulation. M) Schematic of the co‐culture assay was shown on the left panel, while the *CD86* mRNA expression was shown on the right panel. Data are presented as mean ± SD. n.s.: not significant, *p < 0.05, **p < 0.01, ^***^ *p* < 0.001, and ****p < 0.0001, ^##^ *p* < 0.01, and ^####^ *p* < 0.0001. Student's t‐test for (C and H); one‐way ANOVA for (D,J); two‐way ANOVA for (E,F, K–M).

Cell‐cell interactions and crosstalk between FLSs and macrophages have been demonstrated to initiate joint inflammation,^[^ ^26^ ^]^ and the increase in M1 macrophages in the synovium prompted us to investigate how resident synovial cells signaled macrophages to enter the joint during DOA. Notably, we found that macrophage activation was accompanied by enhanced glycolytic metabolism in the FLSs of DOA patients, compared to those of OA and normal synovium (**Figure** **2**A). In support of this, several glycolytic genes, including *TXNIP*, *SLC2A1 (GLUT1), SLC2A5, HK1, PFKM (PFK1), FBP1, GAPDH, ENO1, PKM*, and *LDHA* were altered in both the sublining and lining FLSs of the synovium in DOA (Figure 2B; Figure S6, Supporting Information). Furthermore, the expression of TXNIP, which we and others have previously shown to promote the internalization of glucose transporter GLUT1 and thus regulate glycolysis,^[^ ^27^ ^]^ was downregulated in the synovium of DOA patients compared with OA patients (Figure 2C). Accordingly, the expression of GLUT1 was elevated in the synovium of DOA patients (Figure S7A, Supporting Information). Next, we sought to examine whether chronic low‐grade inflammation induced by the DOA environment affected the mRNA expression of *TXNIP* and the subsequent glycolytic metabolism of FLSs in vitro. As expected, IL‐1β treatment significantly suppressed the *TXNIP* mRNA expression in FLSs (Figure 2D). Consistent with previous findings that cytokine has a direct effect on the regulation of glycolysis,^[^ ^28^ ^]^ we found that IL‐1β significantly increased the extracellular acidification rate (ECAR) associated with glycolysis and the glycolytic capacity of FLSs (Figure 2E,F). Next, FLSs were cultured with inflammatory stimuli combined with different glucose levels, and the results revealed that a combination of high glucose with IL‐1β could further enhance the glycolysis in FLSs (Figure 2E,F; Figure S7B, Supporting Information), indicating that metabolic changes can interact with cytokine signaling to contribute to joint inflammation in DOA.

Enhanced glycolysis in synovial cells has been demonstrated to be fundamental for providing energy for cytokine production.^[^ ^13^, ^16^ ^]^ GO analysis of the scRNA‐seq data did reveal an increase in inflammatory signaling in the synovium of DOA (Figure 2G). Consistently, the suppression of TXNIP by IL‐1β not only led to an increase in the glycolytic activity, but also facilitated the expression of a series of inflammatory mediators, including *ICAM‐1*, *CCL2*, *MMP13*, and *MMP1* (Figure 2H). We have previously demonstrated that an increase in the expression of ICAM‐1 in regional synovial cells facilitates the recruitment of macrophages into the joint in the T2DM rat model.^[^ ^16^ ^]^ Consistently, cell‐cell contact analysis by CellChat^[^ ^29^ ^]^ revealed that macrophages were the most activated cell type in DOA, and the ICAM signaling during macrophage‐synovial cell interactions was strengthened in the DOA group (Figure S8, Supporting Information), which supports the hypothesis that elevated ICAM‐1 expression in the synovium may contribute to the regulation of macrophages. In addition to ICAM‐1, previous studies have suggested that VCAM‐1, e‐selectin (SELE), and p‐selectin (SELP) are other important adhesion molecules that mediate monocyte transmigration.^[^ ^30^ ^]^ In our scRNA‐seq results, VCAM‐1 was identified as the most enriched gene in the synovium of DOA (Figure S9A, Supporting Information). In addition, the average expression of VCAM‐1 was higher in synovial cells of DOA when compared to that of OA and normal synovial cells (Figure S9A,B, Supporting Information). Consistently, the activation of FLSs or chondrocytes (CHON) by IL‐1β or TNF‐α, either alone or in combination with a high dose of glucose, induced ICAM‐1 and VCAM‐1 expression and thereby facilitating the adhesion of THP1 cells to the activated cells (Figure 2I,J; Figures S9C and S10A,B, Supporting Information). In contrast, inhibiting glycolysis with glucose analog 2‐deoxy‐D‐glucose (2‐DG) significantly reduced *ICAM‐1* mRNA and protein expression, subsequently leading to a decrease in the migration of macrophages (Figure 2K,L; Figure S10C,D, Supporting Information). Next, we investigated whether the increase in glycolysis in synovial cells could modulate the polarization of macrophages using a co‐culture model (Figure 2M). Notably, inhibiting IL‐1β‐stimulated FLSs glycolysis with 2‐DG suppressed the expression of *CD86* mRNA in phorbol 12‐myristate 13‐acetate (PMA)‐induced THP‐1‐differentiated macrophages via a paracrine manner (Figure 2M, right panel). Moreover, the stimulation of THP‐1‐differentiated macrophages by LPS and IFN‐γ significantly increased the *CD86* mRNA expression, and treatment with 2‐DG also dramatically suppressed its expression (Figure S10E,F, Supporting Information). Furthermore, a positive correlation was validated between glycolytic level (represented by GLUT1 expression) and M1 macrophage polarization (iNOS) in the synovium of normal, OA, and DOA groups (Figure S10G, Supporting Information). These results suggest that enhanced glycolysis in synovial cells supports energy production and provides glycolytic intermediates for cytokines production, thereby mediating the recruitment and activation of macrophages within the joint in DOA.

### 2.3 YAP1 Overexpression Drives M1 Macrophage Infiltration via Enhancing Synovial Glycolysis in the DOA Synovium

**Figure 3:**
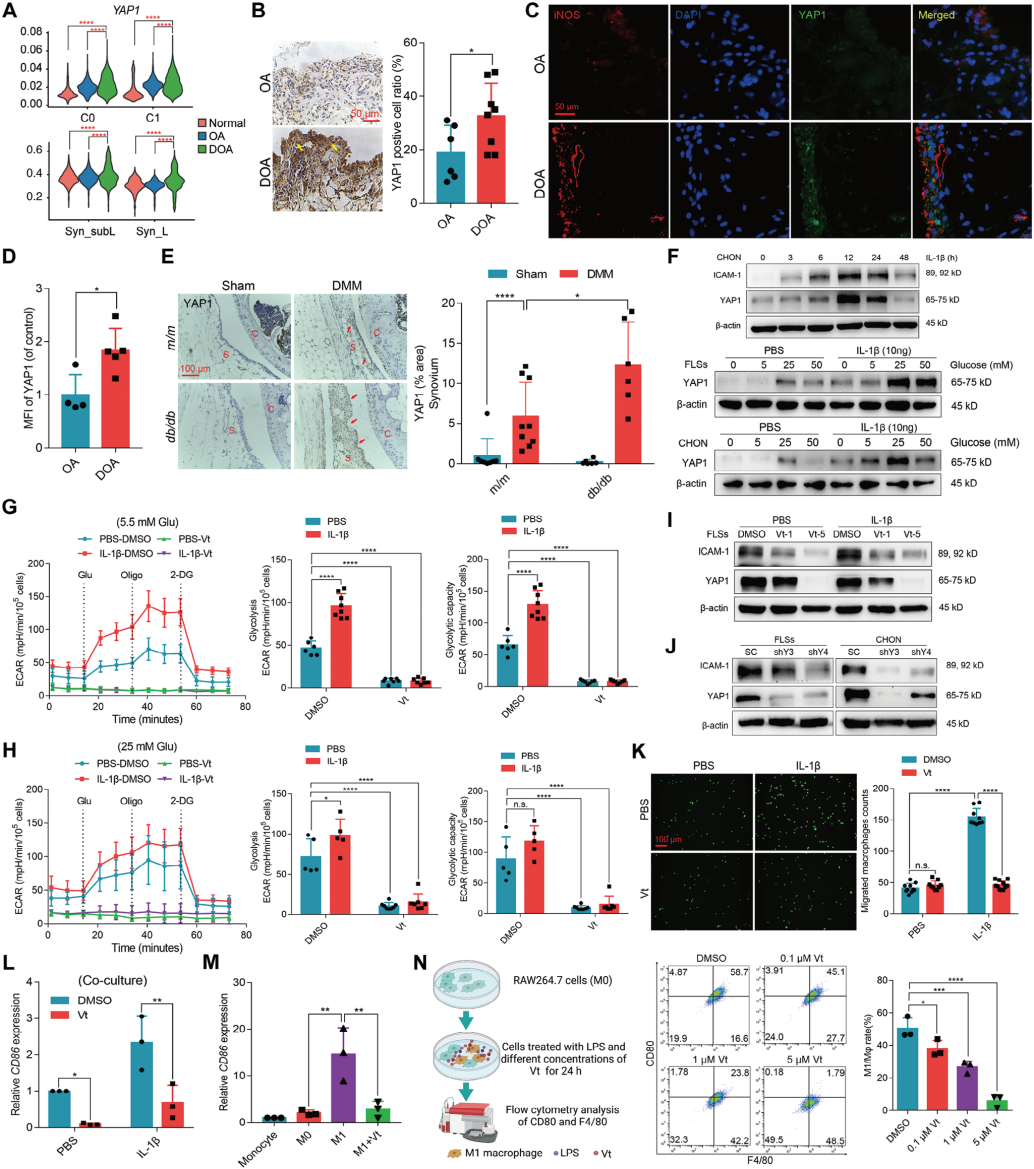
YAP1 regulates the infiltration and M1 macrophage polarization partially through enhancing synovial glycolysis and the expression of adhesion molecules. A) Violin plots of the *YAP1* expression levels in macrophages and FLSs isolated from the synovium of lining and sublining layers. B) Representative images of IHC staining and the quantification of YAP1^+^ cells in the synovium of OA and DOA patients (*n* ≥ 7). C,D) Representative images of IF staining C) and quantification of YAP1^+^ cells in the synovium of OA and DOA patients D) (*n* ≥ 4). E) Representative images of YAP1 IHC staining and quantification of YAP1^+^ cells in the indicated groups (*n* = 6). F) Immunoblotting of ICAM‐1 and YAP1 expression in FLSs and CHON after treatment with IL‐1β for different time points (0, 3, 6, 12, 24, and 48 h) or by different glucose concentrations (0, 5.5, 25, and 50 mm), along with PBS or IL‐1β for 24 h. G,H) Seahorse analysis of ECAR, glycolysis, and glycolytic capacity in the FLSs treated with different glucose concentrations (5.5 or 25 mm) and then with PBS or IL‐1β and DMSO or Vt for another 24 h. I) Immunoblotting of ICAM‐1 and YAP1 expression in the FLSs treated with PBS, or IL‐1β, and DMSO or Vt of different concentrations (1 or 5 µm) for 24 h. J) Immunoblotting of ICAM‐1 and YAP1 expression in FLSs and CHON after infected with lentivirus expressing SC, shY#3, or shY#4 for 48 h, followed by the treatment with PBS or IL‐1β for another 24 h. K) FLSs were treated with PBS or IL‐1β and DMSO or Vt for 6 h, and then the fluorescent THP‐1 cells were added into the well of FLSs for 1 h, to determine the monocyte adhesion. L) For the co‐culture assay, FLSs were first treated with IL‐1β and Vt for 24 h, and then the FLSs and THP‐1 were co‐cultured for another 24 h, followed by the determination of *CD86* mRNA expression. M) Vt inhibits *CD86* mRNA expression. THP‐1 cells were cultured with PMA for 6 h, and then treated with LPS and IFN‐γ, and Vt for another 24 h, and the qPCR was applied to exam *CD86* mRNA level. N) Schematic of flow cytometry assay. RAW264.7 cells were treated with LPS and Vt for 24 h, and then the abundance of CD80 and F4/80 were measured by flow cytometry. Data are presented as mean ± SD. n.s.: not significant, *p < 0.05, **p < 0.01, and ****p < 0.0001. Student's t‐test for (B,D); one‐way ANOVA for (M,N); two‐way ANOVA for (E,G,H,K,L).

YAP1 is a vital mechanotransduction mediator for OA onset and progression.^[^ ^31^ ^]^ We and others have recently demonstrated that YAP1 is also involved in regulating cellular senescence, metabolic reprogramming, and inflammation during the initiation of surgery‐induced OA.^[^ ^32^ ^]^ Herein, we found that the expression of YAP1 and its up‐ and downstream molecules was altered in a series of cells, including macrophages, synovial cells, pericyte, and ECs, as shown in violin plots of the scRNA‐seq data (**Figure** **3**A; Figure S11, Supporting Information). Consistently, IHC and immunofluorescence (IF) staining validated that YAP1 was overexpressed in the synovium of DOA patients relative to OA patients (Figure 3B–D). Similar results were obtained in the *db*/*db* mice subjected to DMM surgery compared to control mice (Figure 3E; Figure S12A, Supporting Information). Given that the aforementioned data demonstrated that high glucose combined with IL‐1β promoted the FLSs glycolysis, whether the metabolism of FLSs could be regulated by YAP1 remains elusive. Similar to the expression pattern of ICAM‐1, stimulation of FLSs or CHON with IL‐1β, alone or in combination with different concentrations of glucose, dose‐dependently increased the expression of YAP1 (Figure 3F). We have recently demonstrated that TXNIP is negatively regulated by YAP1 in several cell types,^[^ ^27a^ ^]^ herein, inhibiting YAP1 with Verteporfin (Vt) could mitigate IL‐1β‐induced TXNIP suppression in FLSs (Figure S12B, Supporting Information). Accordingly, Vt significantly reduced the glycolysis of FLSs triggered by different concentrations of glucose and IL‐1β which mimic the diabetic environment in vitro (Figure 3G,H; Figure S12B, Supporting Information).

On the other hand, we also evaluated the expression of ICAM‐1 in response to YAP1 suppression. Notably, YAP1 blockade with Vt dose‐dependently decreased IL‐1β‐induced ICAM‐1 expression (Figure 3I; Figure S12C, Supporting Information). Consistently, knockdown of YAP1 using small hairpin RNAs (shRNAs: shY#3 and shY#4) also reduced the expression of ICAM‐1 following stimulation with IL‐1β or TNF‐α plus high glucose in the activated FLSs and CHON (Figure 3J; Figure S12D,E, Supporting Information). In contrast, transient transfection of plasmids encoding HA‐YAP1 or constitutively activated HA‐YAP1‐S5A into CHON further increased IL‐1β‐induced ICAM‐1 expression (Figure S12F, Supporting Information). Furthermore, macrophage migration was impaired when FLSs were treated with Vt in the presence of IL‐1β in vitro (Figure 3K). Since YAP1 has been reported to regulate M1/M2 macrophage polarization in inflammatory bowel disease,^[^ ^33^ ^]^ we then examined whether YAP1 could regulate synovial inflammation by orchestrating macrophage polarization using a co‐culture system. Notably, FLSs treated with IL‐1β elevated the *CD86* mRNA expression in THP‐1‐differentiated macrophages, whereas was abolished by Vt treatment (Figure 3L), implying that YAP1 modulates FLSs to macrophages interaction via a paracrine manner. In addition, Vt directly suppressed the mRNA expression of *CD86* in THP1‐derived M1 macrophages (Figure 3M). Likewise, YAP1 inhibition by Vt markedly decreased the M1 macrophage polarization in RAW264.7 cells (Figure 3N). These results suggest that YAP1 is a key driver of synovial glycolysis, and targeting YAP1 can mitigate glycolysis thereby attenuating macrophage recruitment and polarization.

### 2.4 The Characteristics of YAP1 Inhibitor‐Loaded M1 Membrane‐Coated Nanoparticles

Currently, the role of YAP1 in cartilage remains controversial. Some studies have found that depletion of YAP1 attenuates cartilage destruction during OA,^[^ ^34^ ^]^ while other studies have demonstrated that YAP1 depletion promotes OA progression.^[^ ^32^, ^35^ ^]^ Although the expression of YAP1 was significantly increased in the cartilage of *db*/*db* DOA mice (Figure S13, Supporting Information), our recent finding that depletion of YAP1 triggers cellular senescence of chondrocytes and cartilage degeneration prompted us to improve the targeting specificity of YAP1 aiming to minimize undesigned side‐effect.^[^ ^35^, ^36^ ^]^

**Figure 4:**
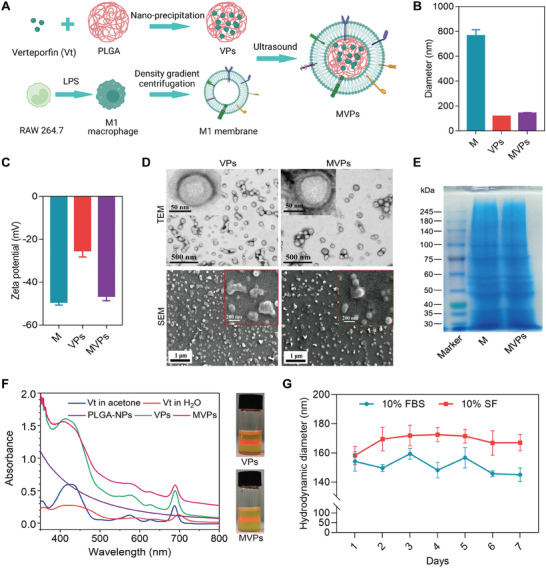
Preparation and characterization of MVPs. A) An illustration depicting the process of preparing MVPs. Briefly, the Vt‐loaded PLGA nanoparticles were prepared by nano‐precipitation, and the MVPs were obtained by ultrasound after mixing with membrane material derived from LPS‐induced RAW 264.7 macrophage cells. B) The hydrodynamic diameters of M, VPs, and MVPs. C) The zeta potential, which indicates the surface charge of M, VPs, and MVPs, was determined. D) Transmission electron microscopy (TEM) (up) and scanning electron microscopy (SEM) images (down) of VPs and MVPs were obtained. E) SDS‐PAGE (sodium dodecyl sulfate‐polyacrylamide gel electrophoresis) analysis of the protein composition of membrane (M) and MVPs, respectively. F) The UV–vis spectra analysis of Vt in water/acetone, PLGA‐NPs, VPs, and MVPs, respectively. G) The hydrodynamic diameter changes of MVPs at different times in PBS with 10% FBS or 10% synovial fluid (SF).

Macrophage membrane‐coated nanoparticles have been widely used in inflammatory diseases including arthritis,^[^ ^37^ ^]^ owing to their superior biocompatibility, prolonged circulation, and disease‐relevant targeting abilities.^[^ ^38^ ^]^ Therefore, we aimed to increase the targeting specificity of Vt using macrophage membrane‐camouflaged nanoparticles, which can improve tissue retention and drug delivery efficiency.^[^ ^19^ ^]^ As illustrated in **Figure** **4**A, the nanoparticle cores consisted of Vt‐loaded PLGA nanoparticles (VPs) that were prepared via nanoprecipitation. Macrophage‐derived membranes were then coated onto VPs by sonication to generate MVPs. Of note, the loading efficiency of VPs and MVPs were calculated to be 0.56% and 0.27%, whereas the encapsulation rate was 96.51% and 91.52%, respectively (Figure S14A, Supporting Information). An optimized ratio of membrane (M) to VPs of 1:1.5 was determined by measuring the hydrodynamic diameter in PBS (Figure S14B, Supporting Information). The increase of ≈26 nm in the hydrodynamic diameter of the MVPs compared to the VPs demonstrated that the macrophage membrane was successfully coated onto the surface of the nanoparticles (Figure 4B). Moreover, the zeta potential of MVPs was found to be less negative compared to that of the cores but was similar to that of the macrophage membrane (Figure 4C), strongly evidenced by the successful coating of the macrophage membranes.^[^ ^39^ ^]^ MVP structure was then visualized by transmission electron microscopy (TEM) after phosphotungstic acid staining, to reveal the internal structure of the nanoparticles. Of note, the typical spherical core‐shell structure of the MVPs was evident in the TEM images, and additionally, there were microfolds on the surface of the MVPs (Figure 4D, upper), which explained the slightly greater thickness of the MVPs compared to the cell membrane. According to the scanning electron microscope (SEM) images, MVPs remained circular dispersed spheres, while some VPs lost their regular spherical shape and fused, indicating the superiority of MVPs for stability (Figure 4D, lower). Sodium dodecyl sulfate‐polyacrylamide gel electrophoresis (SDS‐PAGE) confirmed the presence and enrichment of key surface antigens derived from the macrophage membrane onto the nanoparticles (Figure 4E). Furthermore, the dispersity of Vt in a series of nanoparticles was evaluated. As expected, Vt in acetone, VPs, and MVPs exhibited robust optical absorption at 689 nm, while Vt in water had a redshift of 10 nm, demonstrating that Vt was uniformly dispersed in PLGA cores. Moreover, VPs and MVPs exhibited good dispersion in aqueous solutions with the Tyndall effect (Figure 4F, right panel), reflecting remarkable colloidal stability. VPs and MVPs also showed a sustained‐release effect of Vt (Figure S14C, Supporting Information). MVP sizes remained stable in PBS with 10% FBS or 10% synovial fluid (SF) over 7 days (Figure 4G). Collectively, these comprehensive characterizations verify the successful development of MVPs as stable and efficient Vt delivery vehicles.

### 2.5 Synovial Cellular Uptake, Joint Retention, and Distribution of the MVPs

**Figure 5:**
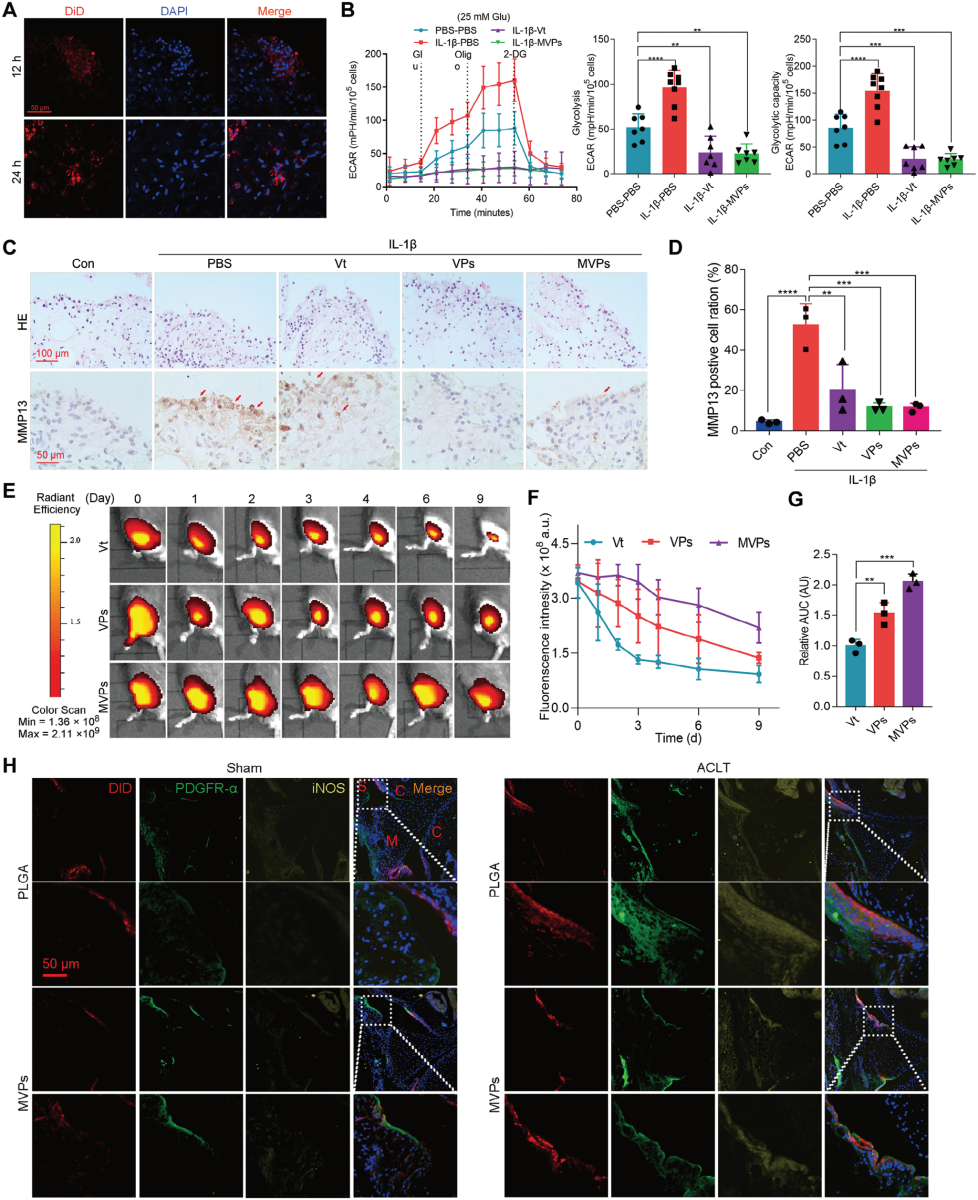
Synovial cellular uptake, joint retention, and distribution of MVPs. A) Representative fluorescence images of human synovial explants treated with DiD‐labeled MVPs (red) for 12 or 24 h. B) Seahorse analysis of ECAR, glycolysis, and glycolytic capacity in the FLSs treated with PBS, Vt, or MVPs with or without the stimulation by IL‐1β for 24 h. Representative images of HE and IHC staining C) and quantification of MMP 13^+^ cells in the synovial explants treated with PBS, Vt, or MVPs with or without the stimulation with IL‐1β for 8 days D) (*n* = 3). E–G) Joint retention of Vt, VPs, and MVPs. Representative images of the knee joints over 9 days after an intra‐articular injection of DiR‐labeled Vt (5 µm), VPs (5 µm), and MVPs (5 µm), (E); Quantitative analysis of time course radiant efficiency within knee joints after intra‐articular injection of DiR‐labeled Vt, VPs, and MVPs, and the area under the curve (AUC) based on fluorescence intensity profile F,G) (*n* = 3). H) Representative fluorescence images of DiD‐labeled MVPs (Red), along with synovium marker PDGFR‐α (green) and macrophage marker iNOS (yellow) in mice knee. The 10‐week‐old *db/db* mice that received sham or anterior cruciate ligament specificity (ACLT) surgery were treated with intra‐articular injections of DiD‐labeled PLGA or DiD‐labeled MVPs (twice a week). The joints were harvested 6 days later for analyses (*n* ≥ 4). Data are presented as mean ± SD. **p < 0.01, ^***^ *p* < 0.001, and ****p < 0.0001. Two‐way ANOVA for (B), one‐way ANOVA for (D,G).

To assess the biocompatibility of the MVPs, a Cell Counting Kit‐8 (CCK‐8) assay was performed in THP‐1‐differentiated macrophages and FLSs. No obvious cytotoxicity was observed when the cells were treated with the MVPs at concentrations ranging between 5 to 20 µm (Figure S15A,B, Supporting Information). Then, we assessed the cellular uptake of MVPs by macrophages. Of note, the cellular uptake occurred in a dose‐ and time‐dependent manner, ranging from 40 to 200 µg mL^−1^ and 3 to 15 h, with an optimal dose of 150 µg mL^−1^ and an optimal incubation time of 12 h (Figure S15C, Supporting Information). In addition, a stronger 1,1′‐dioctadecyl‐3,3,3′,3′‐tetramethylindodicarbocyanine 4‐chlorobenzene sulfonate salt (DiD)‐MVPs, which were labeled with the red fluorescent dye, in the M1‐polarized macrophages was observed compared to that from M0 and M2 macrophages (Figure S15D, Supporting Information), indicating a superior uptake efficiency of MVPs by M1 macrophage. Accordingly, the MVPs were found to suppress the polarization of M1 macrophages (Figure S15E, Supporting Information). We then were interesting to evaluate the penetration efficiency of MVPs in vitro by treating synovial explants with DiD‐MVPs. Notably, DiD‐MVPs were able to penetrate the synovium within 12 h (**Figure** **5**A). To further determine the regulatory effect of MVPs on synovial explants, tissues were stimulated with IL‐1β and various treatments for 8 days, followed by histological analysis (Figure S16A, Supporting Information). Inhibiting YAP1 (VPs or MVPs) downregulated the expression of its downstream target gene, including Cyr61, at multiple time points (Figure S16B, Supporting Information). Moreover, MVPs could mitigate IL‐1β and high glucose elicited ECAR levels, exhibiting a similar effect to that of Vt treatment (Figure 5B). Furthermore, inhibition of YAP1 by Vt, VPs, or MVPs significantly reduces the percentage of MMP13‐positive cells in the synovial explants (Figure 5C,D), corroborating previous findings that inhibition of YAP1 can regulate synovial inflammation.^[^ ^40^ ^]^

To assess the therapeutic efficacy of MVPs in DOA, we analyzed the retention profiles and biodistribution in the knee joint using 1,1‐dioctadecyl‐3,3,3,3‐tetramethylindotricarbocyanine iodide (DiR) as an indicator in vivo at various time points. Of note, DiR‐Vt decreased significantly by 3 days, while the MVPs enhanced joint retention up to 9 days, indicating the superiority of MVPs over Vt alone (Figure 5E–G; Figure S17A, Supporting Information). The biodistribution of MVPs in joint tissues and internal organs was also examined. At 24 h post‐injection, fluorescence signals accumulated in various knee joint components, including femoral condyles, tibial plateau, and synovium (Figure S17B, Supporting Information). The detection of high fluorescence signals in the livers of mice suggested that MVPs might had higher liver retention (Figure S17C, Supporting Information). To further assess the targeting specificity of MVPs in the synovium, anterior cruciate ligament transection (ACLT)‐induced OA was established for 6 days, followed by DiD‐labeled MVPs injection every three days. Consistent with the previous study,^[^ ^41^ ^]^ ACLT induced synovial hyperplasia by day 6 in the *db*/*db* mice, as demonstrated by the significant increase of platelet‐derived growth factor receptor‐α (PDGFR‐α)‐stained FLSs and iNOS‐stained macrophages, respectively (Figure 5H). Although MVPs are designated to be uptake by M1 macrophages, we observed that the DiD‐labeled MVPs were enriched in the hyperplastic synovium, co‐localizing with both iNOS^+^ and PDGFR‐α^+^ synovial cells (Figure 5H), suggesting that the MVPs could be uptake by the two major cell types (FLSs and macrophages) in the synovium. Significantly, the uptake of MVPs could suppress synovial hyperplasia via inhibition of PDGFR‐α and iNOS‐stained cells (Figure 5H). These results suggest that YAP1 inhibitor‐loaded M1 membrane‐coated nanoparticles are a promising strategy for dual targeting FLSs and M1 macrophages for treating joint diseases.

### 2.6 MVPs Attenuate OA Pain and Joint Destruction

**Figure 6:**
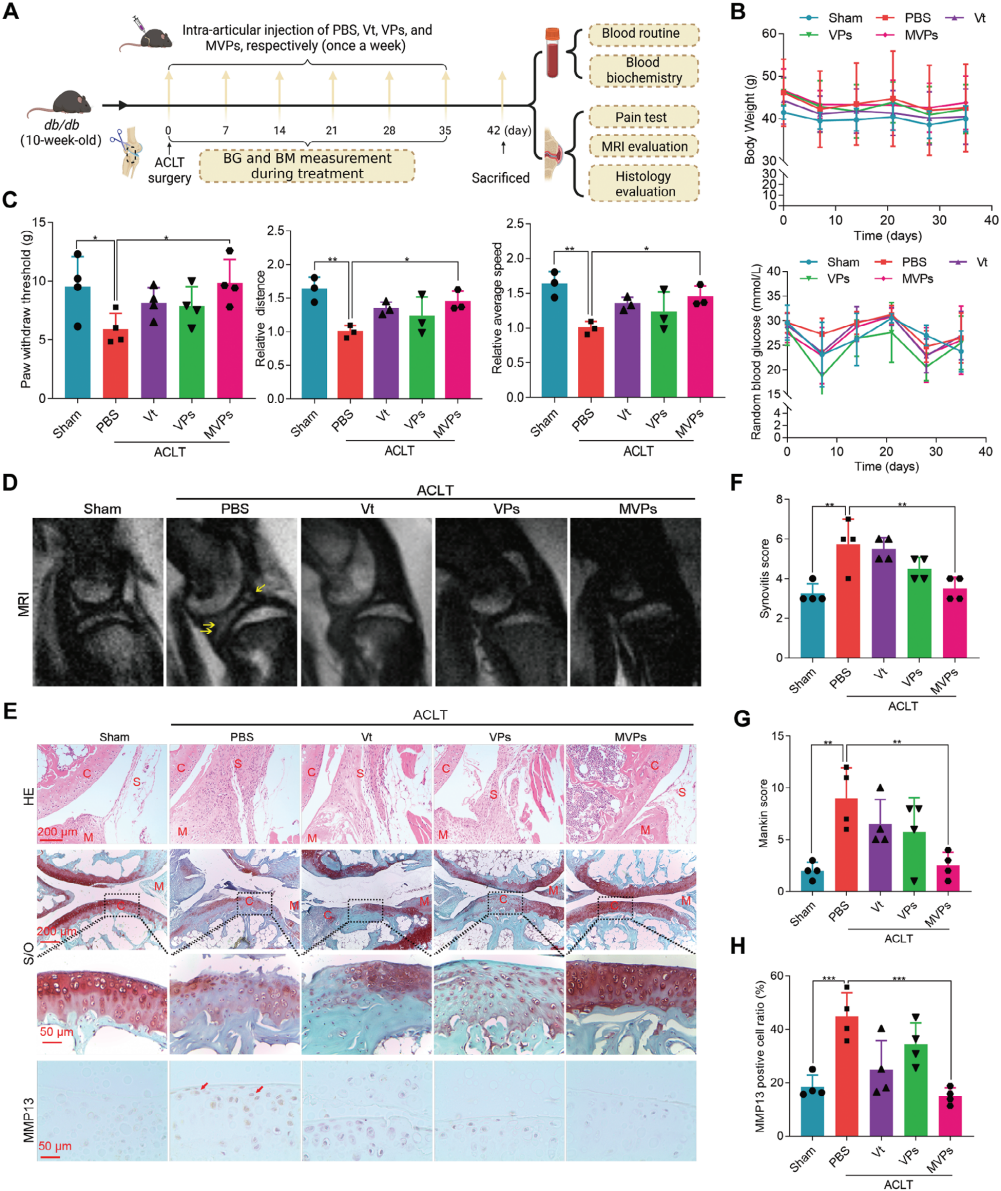
MVPs attenuate joint destruction in ACLT surgery‐induced DOA model. A) Schematic diagram illustrating the establishment of the DOA mice model and the therapeutic evaluation of indicated treatments. In brief, *db/db* mice at 10 weeks of age received sham or ACLT surgery and then were treated with intra‐articular injections of PBS, Vt (5 µm), VPs (5 µm), or MVPs (5 µm) immediately and once every week. The joints were harvested 6 weeks later for further analysis. B) The glucose and weight levels of the mice in the study are monitored over time. C) Pain analysis. The von Frey assay is performed 6 weeks after ACLT surgery. Changes in spontaneous activity, including travel distance and average walking speed, were evaluated using the Laboratory Animal Behavior Observation Registration and Analysis System (LABORAS, Metris, Netherlands) (*n* = 3). D) Representative MRI images of the right knee. E–H) Representative HE images of synovium, Safranin O/Fast Green staining, and IHC staining of knee joints at 6 weeks after surgery (E). The synovitis score was measured (F), the OA severity of knee joints was assessed by Mankin score G), and the quantification of MMP13‐positive chondrocytes H) *n* ≥ 4. Data are presented as mean ±SD. *p < 0.05, **p < 0.01, and ^***^ *p* < 0.001. One‐way ANOVA for (C,F–H).

To explore the function of YAP1 in DOA, DMM surgery was performed in the *db*/*db* diabetic mice, followed by the injection of Vt twice a week for 8 weeks (Figure S18A, Supporting Information). Inhibiting YAP1 with Vt partially alleviated the synovitis and Osteoarthritis Research Society International (OARSI) scores in the OA *db*/*db* mice (Figure S18B,C, Supporting Information). These results suggest that YAP1 may contribute to DOA development and progression, and inhibiting its activity might be a therapeutic strategy for DOA treatment. Next, the functional role of MVPs in DOA was evaluated using a more severe DOA model established by ACLT surgery in 10‐week‐old *db*/*db* mice, followed by a series of treatments (**Figure** **6**A). No overt changes were observed in mouse body weight, random blood glucose, or hematologic parameters (Figure 6B; Figure S19, Supporting Information). HE staining of the heart, liver, spleen, lung, and kidney showed no obvious inflammatory infiltrates or damage, which indicated good biocompatibility of these formulations (Figure S20, Supporting Information). Since pain is the most common symptom of OA and DOA in patients,^[^ ^42^ ^]^ DOA mice pain was monitored by animal behavior analysis. Notably, intra‐articular delivery of MVPs effectively attenuated ACLT‐induced DOA joint pain based on von Frey assays, the Laboratory Animal Behavior Observation Registration and Analysis System, and rotarod assays (Figure 6C). In addition, MVPs significantly reduced joint inflammation and cartilage degeneration in DOA mice by small animal MRI (Figure 6D). HE and Safranin O staining further confirmed that synovial hyperplasia, cartilage damage, surface fibrosis, and proteoglycan loss were induced in the ACLT diabetic mice (Figure 6E,F), and intra‐articular injection of MVPs into ACLT knees greatly attenuated the OA phenotype, with cartilage preservation and reduced synovitis score (Figure 6E,F). Consistent with this finding, MVPs treatment was able to reduce MMP13‐stained cells, suggesting efficacy in impeding OA progression by inhibiting MMP13 activity (Figure 6E–H).

**Figure 7:**
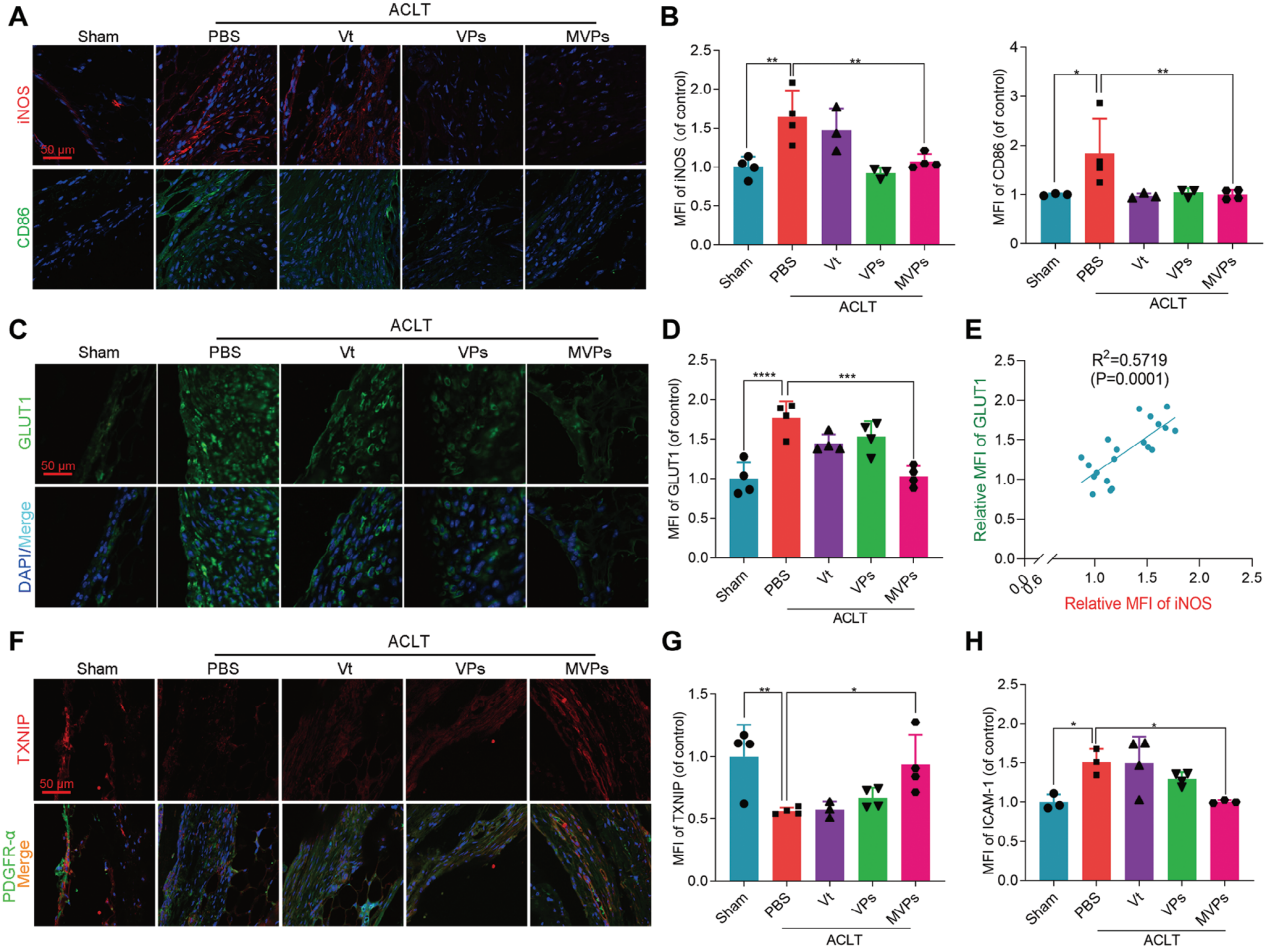
MVPs suppress synovial glycolysis and M1 macrophage infiltration in vivo. A,B) Representative fluorescent staining images of iNOS and CD86 (A), the relative mean fluorescence intensity (MFI) of CD86 in the synovium was quantified in B (*n* ≥ 3). C–E) Representative fluorescent staining images of GLUT1 (C), the relative MFI of GLUT1 in the synovium was quantified (D), and the correlation between GLUT1 and iNOS expression was analyzed in E (*n* ≥ 3). F) Representative fluorescent staining images of ICAM‐1 and PDGFR‐α. F–H) Representative fluorescent staining images of TXNIP and PDGFR‐α (F), the relative MFI of TXNIP in the synovium was quantified (G), and the relative MFI of ICAM‐1 was also quantified in H (*n* ≥3). Data are presented as mean ±SD. *p < 0.05, **p < 0.01, ^***^ *p* < 0.001, and ****p < 0.0001. One‐way ANOVA for (B,D,G,H).

To understand the potential mechanism of the therapeutic effect of MVPs observed in scRNA‐seq data, we performed the IHC and IF staining of M1 macrophage biomarkers (CD86 and iNOS). MVPs significantly decreased the percentage of iNOS‐ and CD86‐stained M1 macrophages in the synovium of DOA mice (**Figure** **7**A,B; Figure S21A,B, Supporting Information). We next explored MVPs regulation of glycolytic metabolism in *vivo*. Consistent with the positive correlation observed in glycolytic level with M1 macrophage polarization, the synovial expression of GLUT1, a primary rate‐limiting glucose transporter of glucose metabolism, which is negatively regulated by TXNIP, was reduced following MVPs treatment (Figure 7C–E). Accordingly, we also observed increased TXNIP expression co‐localizing with the synovial marker PDGFR‐α by IF (Figure 7F,G). Moreover, MVPs substantially attenuated YAP1‐regulated ICAM‐1 expression in DOA mouse synovium (Figure 7H; Figure S21C, Supporting Information). Taken together, these results indicate MVPs‐mediated YAP1 targeting as a promising DOA therapeutic approach, largely by suppressing the FLSs glycolysis and M1 macrophage infiltration (Scheme 1).

## 3 Discussion

Osteoarthritis (OA) is the most prevalent degenerative joint disease that causes pain, limited range of motion, and deformity. We and others have previously demonstrated that metabolic dysfunction plays a critical role in the development and progression of OA.^[^ ^16^, ^43^ ^]^ T2DM is a metabolic disorder increasingly recognized as a major risk factor for OA.^[^ ^44^ ^]^ For example, glucose metabolism dysfunction in T2DM can disrupt the homeostasis of articular cartilage and joint integrity, leading to cartilage destruction.^[^ ^45^ ^]^ Moreover, metabolic alterations associated with T2DM can trigger synovial inflammation, which contributes to cartilage damage.^[^ ^4^, ^11^ ^]^ In support of this, previous studies have reported that DM patients have significantly higher concentrations of IL‐6 and IL‐1β in the synovial fluid than that of non‐DM patients.^[^ ^46^ ^]^ In addition, our group recently revealed remarkably elevated matrix metalloproteinase of MMP‐1, −7, −8, −9, −10, and −12 in DOA relative to OA and healthy synovial fluid.^[^ ^47^ ^]^ Therefore, escalated inflammatory cytokine secretion appears pivotal in DOA pathogenesis. Synovial inflammation is driven by many cell‐cell interactions within the synovium.^[^ ^8^ ^]^ Recently, scRNA‐seq has identified various cell types in normal and OA synovium, including FLSs, ECs, pericytes, macrophages, mast cells, natural killer cells, and B cells.^[^ ^7^ ^]^ Other studies using scRNA‐seq or other transcriptomics have also shown increased macrophage numbers in T2DM compared to non‐diabetic OA synovium.^[^ ^16^, ^48^ ^]^ However, the specific cellular changes and corresponding transcriptional profiles in DOA remain poorly understood. In the current study, we identified eight distinct human synovium cell clusters, including Syn_subL FLSs, Syn_L FLSs, ECs, pericytes, macrophages, dendritic cells, lymphocytes, and mast cells, totaling 83 097 cells. Importantly, the percentage of macrophages was found to be significantly increased in the synovium of humans and animals with DOA (Figure 1).

Our primary findings from scRNA‐seq have revealed significant enrichment of activated macrophages along with elevated FLSs glycolysis in DOA synovium, we thus hypothesized that the regional FLSs might provide additional energy via enhanced glycolysis to drive the infiltration of monocytes/macrophages into the injury site. Several lines of evidence support that glycolysis‐ICAM‐1 signaling in FLSs facilitates macrophage tissue infiltration. First, expression of GLUT1, a primary rate‐limiting glucose transporter of glucose metabolism that is negatively regulated by TXNIP, was elevated in the synovium of DOA patients compared to that of OA and normal patients. Second, enhancement of GLUT‐1‐dependent glycolysis in FLSs in DOA might drive M1 macrophage polarization and infiltration; this is aligned with studies showing glycolysis is fundamental for providing sufficient energy and intermediates for macrophage reprogramming.^[^ ^11^, ^14^ ^]^ In addition, inhibiting glycolysis with 2‐DG markedly prevented M1 macrophage infiltration and polarization. Thirdly, although TXNIP regulates intracellular redox state by inhibiting thioredoxin (Trx), it can directly modulate glucose uptake and glycolysis by promoting GLUT1 endocytosis;^[^ ^27^, ^49^ ^]^ Our unbiased results revealed a crucial role for TXNIP‐mediated glycolysis in DOA, further confirmed by the in vitro experiments where chronic inflammation mimicked by IL‐1β and high glucose suppressed TXNIP expression and enhanced GLUT‐1‐dependent glycolysis in FLSs. Lastly, cytokines and adhesion molecules secreted by chondrocytes or synovial cells are necessary for the recruitment of immune cells into the joint,^[^ ^8b^ ^]^ and we found that at least ICAM‐1, which are elevated in the DOA serum and synovium,^[^ ^16^ ^]^ mediated the infiltration and polarization of M1 macrophages. These findings imply that the enhanced glycolysis of FLSs fuels the filtration and M1 polarization of macrophages, which, in turn, drives the progression of DOA.

YAP1 plays a well‐documented role in mediating mechanical responses and has recently been implicated in the development and progression of OA. For example, the deletion of YAP1 promotes cartilage disruption by interacting with nuclear factor kappa B (NF‐kB) or senescence induction.^[^ ^17^, ^35^ ^]^ Conversely, YAP1 is increased in the cartilage, and suppressing YAP1 attenuates OA progression.^[^ ^34^ ^]^ These discrepancies may stem from variances in animal models, timepoints, and, more importantly, the diverse role of YAP1 in regulating mechanical transduction, cell senescence, and inflammation.^[^ ^33^, ^50^ ^]^ In our study, unbiased scRNA‐seq results revealed a remarkable increase of YAP1 expression in the DOA patients and mouse synovium. We further demonstrated that YAP1 positively regulated glycolysis in FLSs, and inhibiting YAP1 with Vt significantly abolished the increase in glycolysis induced by glucose combined with inflammatory stimuli. These findings were aligned with a recent report showing that YAP1 promotes the accumulation of glycolytic intermediates and metabolites by upregulating GLUT expression during acute pressure overload.^[^ ^32d^ ^]^ In addition, the YAP1/TXNIP signaling axis was shown to be fundamental for modulating GLUT1 endocytosis and subsequent glycolytic changes.^[^ ^27b^ ^]^ In the context of synovial inflammation, emerging evidence has suggested that YAP1 is essential for providing energy to fuel synovial inflammation through glycolysis and high lactate production.^[^ ^51^ ^]^ Since the recruitment and metabolic reprogramming of macrophages are energy‐intensive processes, we, therefore, suggest that the YAP1‐glycolysis‐ICAM‐1 signaling in FLSs plays a role in fueling the macrophage infiltration and M1 polarization, propagating DOA progression. Previous studies have demonstrated that the expression of YAP1 and ICAM‐1 is elevated in the endothelium of diabetic patients.^[^ ^52^ ^]^ Moreover, high glucose and/or IL‐1β robustly stimulate the expression of ICAM‐1 within the endothelial cells, facilitating the recruitment of macrophages entering the local joint.^[^ ^16^, ^53^ ^]^ Our scRNA‐seq data revealed upregulated YAP1 signaling in both pericyte and endothelial cells, suggesting elevated ICAM‐1 level in the endothelium might also promote monocyte/macrophage vascular adhesion, and subsequent joint infiltration.

The above findings suggested that YAP1 is a potential synovium‐specific therapeutic target for DOA; meanwhile, the role of YAP1 in cartilage is still vague; therefore, to increase the targeting specificity of Vt, we developed a drug delivery system using macrophage membrane‐camouflaged nanoparticles to specifically target and regulate the activity of YAP1 in the synovium for DOA treatment. Membrane‐camouflaged drug‐loaded nanoparticles have shown promise in treating inflammatory diseases, including various forms of arthritis. For instance, engineered platelet microparticle, apoptotic chondrocyte, neutrophil‐erythrocyte hybrid, neutrophil, and macrophage membranes have been employed to treat arthritis, including OA.^[^ ^19^, ^54^ ^]^ According to our scRNA‐seq results macrophages were the major altered immune cells in DOA synovium; we utilized macrophage membrane to construct Vt‐loaded nanoparticles. As expected, MVPs exhibited pronounced efficacy in alleviating DOA, as supported by several lines of observations: i) prolonged retention of MVPs in the joint contributed to the inhibition of MMP13 expression; ii) MVPs decreased the pain scores, synovitis scores, OARSI scores, and M1 macrophage polarization in *vivo*, but with marginal effect on blood tests and major organs histology. iii) MVPs demonstrated targeting specificity to synovial FLSs and macrophages, indicating preferential synovial versus chondrocyte uptake in vivo. This aligns with a recent study showing nanoparticles (>143.3 nm like MVPs) cannot penetrate the superficial cartilage or full thickness of cartilage with collagen II (pore size 50–60 nm) and proteoglycan network (20 nm pore size).^[^ ^55^ ^]^ This study represents the first instance of using M1 macrophage membrane‐coated YAP1‐targeted nanoparticles, providing novel insights into targeting macrophages and YAP1 for the attenuation of DOA.

## 4 Conclusion

In summary, we represent the first comprehensive comparative analysis of cell populations in DOA synovium and lay the groundwork for therapeutic targeting of FLSs glycolysis and macrophage reprogramming in DOA. We developed a drug delivery system called an **M** 1 macrophage membrane‐camouflaged **V** t‐loaded **P** LGA nanoparticle **s** (MVPs) to ameliorate DOA progression by suppressing the synovial glycolysis and inhibiting the infiltration and activation of M1 macrophages via the YAP1/TXNIP signaling axis. Moreover, the findings suggest that YAP1 is a potential therapeutic target for DOA treatment, which might shed light on the development of new therapeutic strategies for preventing DOA by fine‐tuning macrophage polarization.

## 5 Experimental Section

### Ethics Statement

Human synovium was isolated from suprapatellar fat that was dissected from the knee joint of OA or ACL injury patients, with the approval of the Human Ethics Committee of the First Affiliated Hospital of Jinan University (KY‐2021‐064, KY‐2021‐022, [2019]‐KY‐001). All patients signed an informed consent form approved by the local institutional review board. All animal experiments were approved by the ethical committee of the Institute of Laboratory Animal Science, Jinan University (IACUC‐20211110‐04, IACUC‐20210412‐02, IACUC‐20181029‐03).

### Cell Culture

THP‐1 cells were purchased from the American Type Culture Collection (ATCC), and cells were cultured in Roswell Park Memorial Institute 1640 (RPMI 1640) with 10% FBS, 100 µg mL^−1^ streptomycin, and 100 U mL^−1^ penicillin. For M1 macrophage polarization, THP‐1 at a density of 1 × 10^5^ cells/well was seeded into the 6‐well plate for 24 h, and then cells were treated with 100 ng mL^−1^ PMA for 6 h, and 100 ng mL^−1^ of lipopolysaccharide (LPS) and 20 ng mL^−1^ of interferon‐γ (IFN‐γ) for another 24 h.

RAW 264.7 cells were cultured in Dulbecco's modified Eagle's medium (DMEM) with 10% heat‐inactivated FBS, 100 µg mL^−1^ streptomycin, and 100 U mL^−1^ penicillin. For M1 polarization, RAW 264.7 cells were treated with 100 ng mL^−1^ of LPS and different concentrations of Vt (0.1, 1, and 5 µm), or Vt (2 µm) and MVPs (2 µm) for 24 h, followed by the flow cytometry analysis of M1 polarization using antibodies against CD80 and F4/80. For M2 polarization, RAW 264.7 cells were treated with 20 ng mL^−1^ of IL‐4 and 20 ng mL^−1^ of IL‐10 for 48 h.

For isolation and culture of primary fibroblast‐like synoviocytes (FLSs), tissues were isolated from the patients receiving total knee arthroplasty (TKA) surgery. Briefly, synovium tissues were incubated with DMEM containing 0.2% type I collagenase (Gibco) for 2 h, followed by 0.25% trypsin‐ethylenediaminetetraacetic acid (EDTA) (Invitrogen) for 30 min. After being filtered through a 70 µm strainer (Fisher Scientific), cells were cultured in DMEM containing 10% Fetal Bovine Serum (FBS, Gibco), 100 µg mL^−1^ streptomycin, and 100 U mL^−1^ penicillin.

For isolation and culture of primary chondrocytes (CHON), CHON was isolated from the patients receiving TKA surgery. Briefly, cartilage tissues were sterile scalpel blade into small pieces (≈2 × 2 mm) incubated with DMEM containing 0.2% type II collagenase (Gibco) for 4 h, followed by 0.25% trypsin‐EDTA (Invitrogen) for 30 min, and after filtered through a 70 µm strainer (Fisher Scientific), cells were cultured in DMEM with 10% FBS, 100 µg mL^−1^ streptomycin, and 100 U mL^−1^ penicillin.

To measure the effects of MVPs on FLSs and macrophages viability, FLSs were treated with different concentrations (0, 100, 200, and 400 µg mL^−1^) of MVPs, the corresponding concentrations of Vt was 0, 5, 10, and 20 µm in MVPs, or RAW 264.7 cells were treated with different concentrations (0, 250, 500, and 1000 µg mL^−1^) of MVPs, the corresponding concentrations of Vt was 0, 12.5, 25, and 50 µm in MVPs, and then the cell viability were measured using CCK‐8 assay.

The Transwell Permeable Supports (Corning) were used in the co‐culture for FLSs and THP‐1 cells. Briefly, FLSs at a density of 6 × 10^5^ cells were seeded into the top insert with 0.4 µm Pore Membrane for 24 h, then treated with or without IL‐1β, 2‐DG, or Vt for another 24 h. THP‐1 cells were seeded at a density of 1 × 10^5^ cells/well into the 24 mm‐bottom plate for 24 h, and then the cells were treated with PMA for 6 h. Add the above insert compartments without medium into a 6‐well plate, and change the fresh medium with 50% DMEM and RIPR 1640; primary FLSs and THP‐1 were co‐cultured for another 24 h.

### ACLT/DMM‐Induced OA and DOA Models

Six‐week‐old male *db/db* mice and the respective background strain m/m mice were purchased from GemPharmatech Co., Ltd. Mice were maintained under specific pathogen‐free rooms in the Institute of Laboratory Animal Science of Jinan University. For the surgically induced OA model, 10‐week‐old mice was anesthetized with 350 and 450 mg kg^−1^ tribromoethyl alcohol concentration in *m/m* and *db/db* mice and then transected the medial meniscotibial ligament (MMTL) to induce the destabilization of the medial meniscus (DMM) associated OA or transected the anterior cruciate ligament (ACL) to establish ACLT‐induced OA model. Sham operations were done on independent mice. To test the therapeutic effect of Vt, VPs, or MVPs, 10 µL PBS, Vt (5 µm), VPs (5 µm), or MVPs (5 µm, indicates the concentration of Vt in 100 µg mL^−1^ of MVPs was 5 µm) was injected intra‐articularly with a 30‐gauge needle into mouse right knees. The first injection was performed immediately after surgery. Injections were then repeated once every week until 6 weeks after the ACLT surgery. All experiments were approved by the local ethical committee.

### Human Subject

Articular synovium samples were sourced from nine patients with DOA (median age of 68.6 years) who underwent TKA. As controls, specimens (median age of 66.3 years) were obtained from nine patients with OA who underwent TKA, and three relatively normal synoviums were collected from those undergoing arthroscopy ACL reconstruction or meniscus repair. Preoperatively, all the patients underwent a knee X‐ray examination, complete blood cell count, and comprehensive metabolic panel. The specimens were used for HE staining, IHC, IF, and scRNA‐seq. Synovial fluid (SF) was obtained from patients who underwent total knee arthroplasty (OA and DOA) surgery. Then, the SF was diluted with PBS for the determination of the stability of MVPs.

### Human Synovial Explant Experiments

Human synovial tissues were isolated from patients who underwent TKA surgery. 5 mm (length) × 5 mm (width) × 2 mm (depth) synovium explants were scissored and cultured in DMEM supplemented with 10% FBS, 1% ITS + Premix, 50 µg mL^−1^ L‐proline, 1% Insulin, 100 µg mL^−1^ streptomycin, and 100 U mL^−1^ penicillin for 2 days. Synovial explants were treated with PBS, IL‐1β in combination with PBS, Vt (5 µm), VPs (5 µm), or MVPs (5 µm), for another 8 days. The final IL‐1β and Vt concentration in the culture medium was 10 ng mL^−1^ and 5 µm, respectively. The medium was replaced every two days. Explants were processed into 6 µm thick paraffin sections for IHC staining. Synovial explants were treated with DiD‐MVPs for 12 or 24 h, and 50 µm thick fresh sections were conducted for IF staining using antibodies against PDGFR‐α and DAPI.

### scRNA‐seq

For scRNA‐seq, the articular synovium was collected as described above. In brief, after washing with PBS, 4–6 grams of synovium tissue were used for further digestion. Compound dissociative enzymes [collagenase I/II/IV and neutral protease (total concentration: 3 mg mL^−1^; ratio: 1:2:2:1) with 10 µg mL^−1^ DNA enzyme] were used to digest human synovial tissues with Miltenyi tissue cell dissociation instrument at 37 °C for 40 min. A complete culture medium (containing 10% FBS) was added to terminate digestion. The digested adipose tissue was filtered through a 70 µm cell strainer, and the pellet was centrifuged at 500 g for 5 min at 4 °C. After the erythrocyte was removed, the single‐cell suspension was resuspended with 15 mL DMEM. Trypan blue staining and an automatic cell counter were used for cell count and activity detection.

Twenty thousand cells were loaded to acquire one single library of 10 000 cells for each sample by Chromium controller (V3 chemistry version, 10X Genomics Inc, San Francisco, USA), barcoded and purified as described by the manufacturer, and sequenced using a 2 × 150 pair‐end configuration on an Illumina HiSeq platform at a sequencing depth of ≈400 million reads. Cell ranger (Version 6.0.2) was used to demultiplex reads, followed by extraction of cell barcode and unique molecular identifiers (UMIs). The cDNA insert was aligned to a reference human genome (GRCh38‐2020).

Seurat package V4^[^ ^56^ ^]^ was used for individual or integrated analysis of datasets. Standard Seurat pipeline was used for filtering, variable gene selection, dimensionality reduction analysis, and clustering. Doublets or cells/nuclei with poor quality (genes > 6000, genes < 200, or >25% genes mapping to mitochondrial genome) were excluded. Seurat Cell‐cycle scoring functions were used to analyze cell proliferation. Proliferative cells were defined as cells in G2/M or S phase. Statistically significant principal components (PC) were calculated, and batch integration was performed using Harmony (version 1.0).^[^ ^57^ ^]^ Uniform manifold approximation and projection (UMAP) were used to present the dimension reduction plots. Different resolutions for clustering were used to demonstrate the robustness of clusters. Sub‐clustering was performed by isolating certain lineage clusters using known marker genes, followed by reanalysis as described above. Differentially expressed genes between normal and radiated groups were identified using FindMarkers within Seurat, and *p* values between them were calculated by “test.use = bimod”. MAGIC was used to impute the drop‐out values for visualization.^[^ ^58^ ^]^ Gene ontology analysis and Gene Set Enrichment Analysis (GSEA) were performed using the ClusterProfiler package.^[^ ^59^ ^]^ Gene Set Variation Analysis (GSVA) was performed using the GSVA package.^[^ ^60^ ^]^ MacSpectrum was used to define Macrophage Polarization Index (MPI) and Activation induced Macrophage Differentiation Index (AMDI). Higher MPI value suggests higher inflammatory features. Higher AMDI value suggests more maturity in macrophage terminal differentiation.^[^ ^22^ ^]^

### Micro‐Computed Tomography (Micro‐CT)

Mouse joints were fixed in 4% paraformaldehyde for 24 h and analyzed by high‐resolution micro‐CT. The study reconstructed and analyzed images using NRecon v1.6 and CTAn v1.9, respectively. 3D model visualization software, CTVol v2.0, was used to analyze the parameters of the trabecular bone in the metaphysis. The scanner was set at a voltage of 50 kVp, a current of 200 µA, and a resolution of 5.7 µm per pixel. Cross‐sectional images of the tibiae subchondral bone were used to perform a 3D histomorphometric analysis. 3D structural parameters analyzed included: BV/TV: trabecular bone volume per tissue volume, Tb. Th: trabecular thickness.

### Quantitative Real‐Time PCR (qPCR)

The total RNA of cartilage and subchondral bone was extracted using TRIzol Plus (Takara Technologies), and 1 µg of RNA was then reverse transcribed to cDNA using the High‐Capacity cDNA Reverse Transcription Kit (Invitrogen) according to the manufacturer's protocol with minor modifications. The primers for *TXNIP*, *ICAM‐1*, *CD86*, *CCL2*, *MMP13*, and *MMP1* amplification were listed as follows in Table S3 (Supporting Information). For qPCR, One Step Real‐Time PCR (Applied Biosystems) was performed by using Fast SYBR@GREEN Master Mix (Life Technologies). All real‐time PCR reactions were performed in duplicate with at least three independent experiments, and the relative expression of each gene was normalized to *GAPDH*, and calculated using the comparative 2^−ΔΔCT^ method.^[^ ^36^ ^]^

### Seahorse XF96 Glycolysis Stress Assay

Extracellular acidification rate (ECAR) was measured by using an XF96 extracellular flux analyzer (Seahorse Bioscience, Billerica, MA, USA). Briefly, FLSs at a density of 1 × 10^4^/well were seeded in 96‐well Seahorse XF96 culture plates with 10% FBS DMEM for 24 h and treated with different concentrations of glucose (5.5, 25, and 50 mm), with or without Vt or MVPs for another 24 h. The protocol followed was done as described in the previous studies.^[^ ^61^ ^]^ All seahorse flux data were normalized to cell numbers measured by the cell counter machine.

### Immunoblotting Analysis (IB)

Immunoblotting was generally carried out as described in the previous studies.^[^ ^62^ ^]^ The primary antibody of TXNIP (1:2000) was purchased from Abcam, the other primary antibodies were obtained from Cell Signaling Technology and were listed below: ICAM‐1 (1:1000), YAP1 (1:1000), P‐YAP1 (1:1000), Cyr61 (1:1000), and VCAM‐1 (1:1000). Secondary HRP‐conjugated antibodies (1:1000 – 1:3000) used for IB analysis were purchased from Cell Signaling Technology (CST, Beverly, MA, USA).

### Infection of FLSs or CHON by Lentivirus

The coding region of human YAP1 was amplified and subsequently subcloned into an LV5‐CMV‐GFP‐EF1a‐Puro lentiviral vector (GenePharma). The different interfering sequences of YAP1 were listed: 5′‐CCTTAACAGTGGCACCTATCA‐3′ (shYAP#3), 5′‐GGTGATACTATCAACCAAAGC‐3′ (shYAP#4). FLSs or CHON were infected by applying viral supernatant (MOI = 100) in 1 mL serum‐free medium for 24 h and then were cultured in the completed medium for further experiments. Plasmids encoding HA‐YAP1 or constitutively activated HA‐YAP‐S5A were constructed as described in the previous studies.^[^ ^32c^ ^]^

### Monocyte Adhesive Assay

To analyze the role of high glucose and inflammation on monocyte adhesion, FLSs or CHON were seeded in 6‐well culture plates at a density of 2 × 10^5^/well with different concentrations of glucose (5.5, 25, and 50 mm) for 24 h, and treated with PBS, IL‐1β, or TNF‐α for another 6 h. To analyze the effect of Vt on high glucose and IL‐1β‐induced enhanced monocyte adhesion, FLSs were treated with different concentrations of glucose (5.5, 25, and 50 mm) for 24 h, and treated with dimethyl sulfoxide (DMSO), or VP for another 18 h, and then treated with PBS, IL‐1β, or TNF‐α for another 6 h. Meanwhile, THP‐1 cells were treated with fluorescent BCECF AM for 1 h, followed by a centrifuge with 500 g, and washed twice with PBS, then 1 × 10^5^ THP‐1 were seeded into each treated well. After incubation for 1 h, the plate was washed twice with PBS and the fluorescent cell count was measured by fluorescence microscope.

### Synthesis and Characterization of MVPs

Briefly, M1 polarized macrophages were harvested and resuspended in ice‐cold PBS, and the membrane was further separated from the cell lysate by subsequent differential and density centrifugation with a regular tabletop microcentrifuge using Membrane Protein Isolation and Cell Fractionation Kit (Invent) according to the manufacturer's protocol. The purified macrophage membrane was finally dissolved in 0.2 mm EDTA buffer, and the protein content was determined by BCA protein assay.

PLGA was used to prepare the model nanoparticle carrier. Vt was dissolved in acetone at a concentration of 10 mm for subsequent use. VPs were prepared by a nanoprecipitation process. PLGA solution (1 mL, 12 mg mL^−1^ in acetone) was mixed with 100 µL Vt, and then the resulting mixture was added rapidly to sterile deionized water (3 mL), followed by evaporating the acetone in a vacuum for 12 h. MVPs were accomplished by ultrasonic method. The dispersion of the M1 macrophage membrane was first sonicated in a bath sonicator (SCIENTZ, SB‐5200DTD) at a frequency of 40 kHz and an output power of 100 W for 5 min. Following by mixing with VPs dispersion (mass ratio of membrane proteins to VPs kept constant at 1:1.5), the resulting mixture was sonicated in 2 min to form MVPs. The ultrasound process was performed under ice bath conditions. The final concentration of MVPs was ≈4 mg mL^−1^ with 200 µm Vt. The hydrodynamic diameter and the zeta potential of nanoparticles were performed using a Nanoparticles Analyzer (HORIBA SZ‐100Z, Japan). The morphologies of bare VPs and MVPs were examined with a transmission electron microscope (TEM, FEI Talos, F200X) after negative staining with 0.2% wt phosphotungstic acid. Scanning electron microscope (SEM, Hitachi, Japan) was also used to investigate their microstructure of them. To evaluate the dispersion of Vt in different systems, the UV–vis absorption spectra of Vt in acetone, Vt in water, PLGA‐NPs, VPs, and MVPs were obtained by scanning the optical characteristics in the range of 350–800 nm vis a UV‐1800 spectrophotometer (Shimadzu, Japan). To determine the encapsulation rate, the VPs and MVPs were centrifuged at 10,000 rad min^−1^ for 30 min, followed by removing the supernatant, and the precipitate was collected and lyophilized. The precipitate was then dissolved in DMSO to measure the fluorescence value in 690 nm by Fluorescence spectrophotometer. To generate a standard curve, Vt in DMSO from 0 to 10 µm in triplicate was also measured. The stability of MVPs was further tested in the media of PBS with 10% FBS or 10% SF. The hydrodynamic diameter was assessed each day during the 7‐day incubation.

### Cellular Uptake Assay

The cellular uptake efficiency and the localization of nanoparticles in macrophages were measured using confocal laser scanning microscopy (CLSM) and flow cytometry (FACS). MVPs were embellished with DiD (red fluorescence dye). The cells were seeded into confocal dishes and 6‐well plates and then treated with different concentrations of DiD‐labeled MVPs (20, 50, 100, 150, and 200 µg mL^−1^), or predetermined times (3, 6, 9, 12, and 15 h) with a 100 µg mL^−1^ DiD‐labeled MVPs. The uptake efficiency of DiD‐labeled MVPs by M0, M2 and M1 macrophages was also measured using CLSM after fixation with 4% paraformaldehyde. Briefly, RAW264.7 cells were used to induce M1 or M2 macrophages polarization, and then the M0, M1, and M2 macrophages was cultured with medium containing DiD‐labeled MVPs (150 µg mL^−1^) for 12 h.

### Circulation Lifetime and Biodistribution of MVPs

The *db/db* mice received ACLT surgery were randomly divided into three groups (DiR‐Vt, DiR‐VPs, and DiR‐MVPs). Then, the mice were intravenously injected with different formulations (DiR equivalent to 0.1% of Vt, the concentration of DiR was ≈0.05 µm). The fluorescence distribution of the right knees was measured at 0, 1, 2, 3, 4, 6, and 9 days after injection by RF‐6000 fluorescence spectrophotometer (Shimadzu, Japan). For the bio‐distribution of MVPs, organ distribution was also measured accordingly.

### Serum Collection

Blood samples were collected in anesthetized mice and dispensed into tubes. And then, tubes were placed at room temperature; after ≈30 min, the tubes were centrifuged at 3500 rpm for 15 min, and the serum was aliquoted into cryotubes for immediate storage at −80 °C until analysis. Levels of aspartate aminotransferase (AST), alanine aminotransferase (ALT), creatinine (CREA), glucose (GLU), and glycated serum protein (GSP) were quantified by an automated chemistry analyzer (Indiko Plus) specific for the detection of animal serum.

### Mechanical Sensitivity (Von Frey) Test

Six mice were placed in different separated cages at once. The bottom of the cage was a wide gauge, wire mesh surface. Animals were allowed to adapt to the environment, including an elevated mesh platform for 20 min. The Von Frey filaments were applied from the underside of the mesh to the plantar surface of the mouse's hind paw. At the threshold, the mouse responded by flicking its paw away from the stimulus and getting the value from the machine until stimulation elicited a hind paw withdrawal; the value was the bearing force.

### Animal Spontaneous Behavior

The assessment of spontaneous behavior was measured by the Laboratory Animal Behavior Observation Registration and Analysis System (LABORAS, Metris, Netherlands). Briefly, after the animals were weighed, four platforms were simultaneously used to monitor four mice for 15 h at the same time frame from 6:00 pm to 9:00 am. The distance of locomotion and average speed of locomotion were assessed.

### Small Animal MRI Imaging

The mice, after 6‐week serial injections (once every week) of PBS, Vt, VPs, and MVPs, were anesthetized and scanned in a 9.4 T µMRI developed for imaging of small animals (Biospec Avance III 9.4/20, Bruker Biospin, Ettlingen, Germany) with a gradient strength of 675 mT/m (BGA 12S gradient system) at room temperature. For imaging of the therapeutic efficacy, an off shelve circular polarized volume coil for imaging of the mouse's right knee with an inner diameter of 40 mm was employed. The samples were placed in the isocenter of the magnet with the lateral position of the knee in an orthogonal position to B0.

### Histology and Immunohistochemistry

Mice joints were fixed in 4% paraformaldehyde for 48 h and followed by decalcification in 10% EDTA (Wuhan Servicebio technology, G1105) for 14 to 28 days and dehydrated in increasing concentrations of ethanol, finally embedded in paraffin. Sections (6 µm) were cut from the paraffin blocks and applied to glass slides. The sections were stained by Safranin O/Fast Green dyes and hematoxylin and eosin (HE, Leagene, China), and the specimens were then mounted. For IHC staining, the protocol was applied as previously reported.^[^ ^63^ ^]^ For the frozen section, sections (50 µm) were cut from the blocks and applied to glass slides for IF staining.^[^ ^64^ ^]^ For IF double staining, tyramide signal amplification plus fluorescence Kit was used (Wuhan Servicebio technology, GT1236). Primary antibodies of CD86, MMP13, iNOS, PDGFR‐α, TXNIP, GLUT1, VCAM‐1, and YAP1 were used. All the key resources used in the experimental section were listed as follows in Table S4 (Supporting Information).

### Statistical Analysis

All experiments were performed at least three times, and the data are expressed as the mean ± standard deviation (SD) for the total number of experiments. All statistical analysis was performed using GraphPad Prism 9 Software. Comparison between the two groups was conducted by using Student's t‐test. The significance of the differences among groups’ means was subjected to one‐way ANOVA analysis, followed by Tukey's test. To examine the influence of two categorical independent variables on one continuous dependent variable, two‐way ANOVA was used. p values <0.05 were considered statistically significant.

## Conflict of Interest

The authors declare no conflict of interest.

## Author Contributions

J.Y., S.L., Z.L., and L.Y. contributed equally to this work. H.T.Z., Z.Z., and X.W. supervised the project and evaluated the results. J.Y., S.L., Z.L., and L.Y. performed most of the experiments and interpreted the data. M.L., K.L.T., Q.X., B.Y., and W.T. performed parts of the immunoblotting. R.P. performed flow cytometry experiments. T.G., Y.X., and J.H. performed some of the animal experiments. J.C., K.Z., and X.W. provided some materials and technical help. All authors reviewed and approved the manuscript.

## Supporting information

Supporting Information

Supplemental Table 1

Supplemental Table 2

## Data Availability

The data that support the findings of this study are available from the corresponding author upon reasonable request.
