# Supplementary material for: Targeting YAP1‐regulated Glycolysis in Fibroblast‐Like Synoviocytes Impairs Macrophage Infiltration to Ameliorate Diabetic Osteoarthritis Progression
Source: Adv Sci (Weinh). 2023 Dec 3;11(5):2304617. doi: 10.1002/advs.202304617 (PMC10837355; doi:10.1002/advs.202304617)
Supplement: Supplementary file 1 — Supporting Information [file ADVS-11-2304617-s003.pdf]

ADVANCED SCIENCE
Open Access

Supporting Information

for *Adv. Sci.*, DOI 10.1002/advs.202304617

Targeting YAP1-regulated Glycolysis in Fibroblast-Like Synoviocytes Impairs Macrophage Infiltration to Ameliorate Diabetic Osteoarthritis Progression

Jie Yang, Shanshan Li, Zhenyan Li, Lutian Yao, Meijing Liu, Kui-Leung Tong, Qiutong Xu, Bo Yu, Rui Peng, Tao Gui, Wang Tang, Yidi Xu, Jiaxu Chen, Jun He, Kewei Zhao, Xiaogang Wang, Xiaoying Wang*, Zhengang Zha* and Huan-Tian Zhang**

Supporting Information

Targeting YAP1-regulated glycolysis in fibroblast-like synoviocytes impairs macrophage infiltration to ameliorate diabetic osteoarthritis progression

Keywords YAP1; Fibroblast-like synoviocytes; Glycolysis; Macrophages infiltration; Diabetic osteoarthritis

Jie Yang, Shanshan Li, Zhenyan Li, Lutian Yao, Meijing Liu, Kui-Leung Tong, Qiutong Xu, Bo Yu, Rui Peng, Tao Gui, Wang Tang, Yidi Xu, Jiaxu Chen, Jun He, Kewei Zhao, Xiaogang Wang, Xiaoying Wang, Zhengang Zha, Huan-Tian Zhang

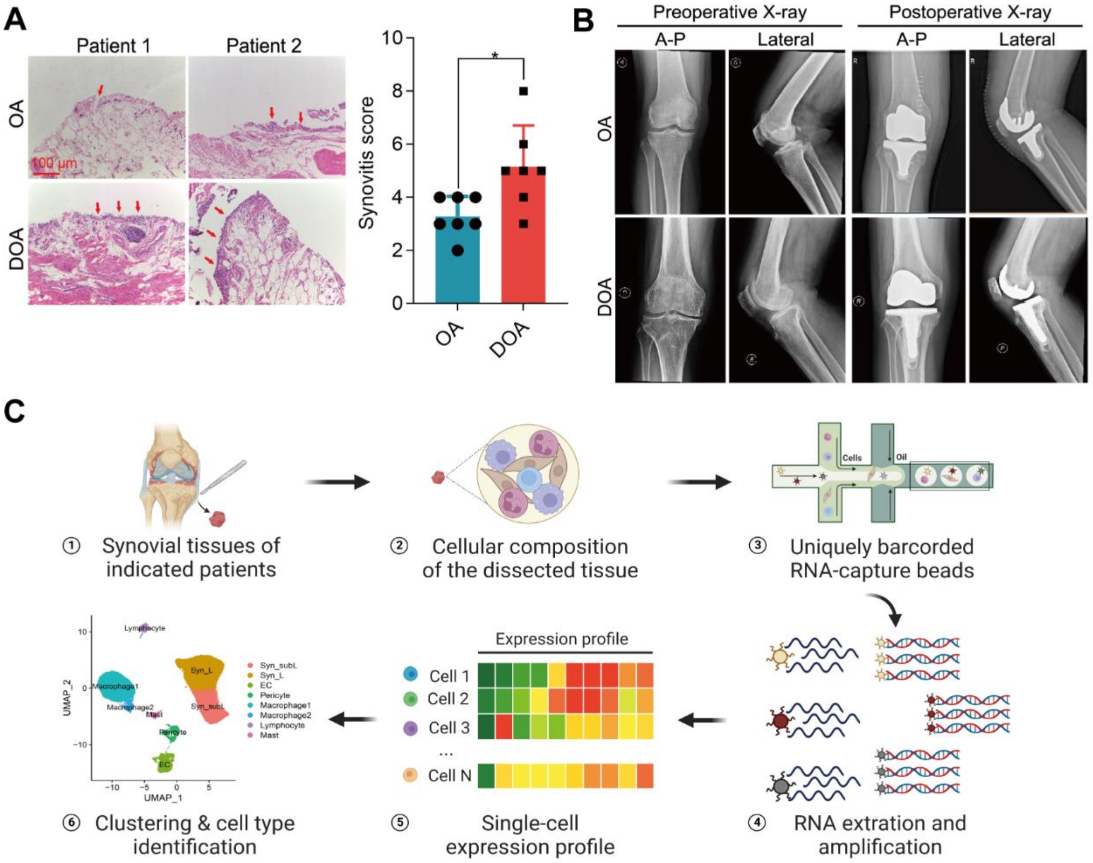

Figure S1. Single-cell RNA sequencing (scRNA-seq) of the indicated synovial specimens. (A) Representative images of HE staining and the quantification of OA and DOA synovitis score. Data are presented as a bar graph with mean ± SD (n = 7). (B) Representative preoperative and postoperative radiographs of patients with knee OA or DOA. (C) Schematic workflow of the experimental strategy for scRNA-seq. *$p < 0.05$. Student's t-test for (A).

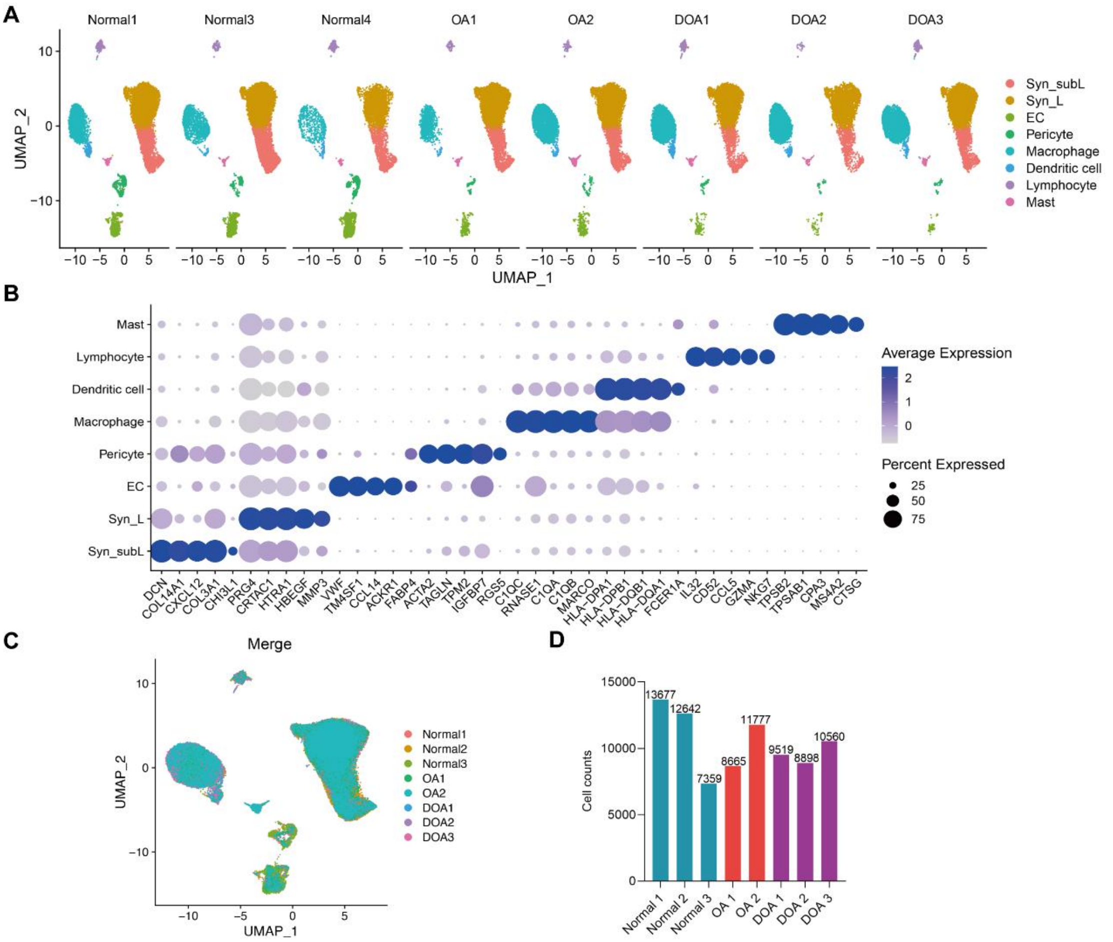

Figure S2. ScRNA-seq identifies several cell clusters in the synovium of normal, OA, and DOA patients. (A) Uniform Manifold Approximation and Projection (UMAP) visualization of the cell types identified in the synovium of normal (n = 3), OA (n = 2), and DOA patients (n = 3). (B) The top 5 differentially expressed markers across the cell types identified in the synovium were shown in the dot plot. (C) The integrated UMAP of the cell types was identified from the synovium of the indicated patients. (D) The total number of cells used for sequencing was marked in each group.

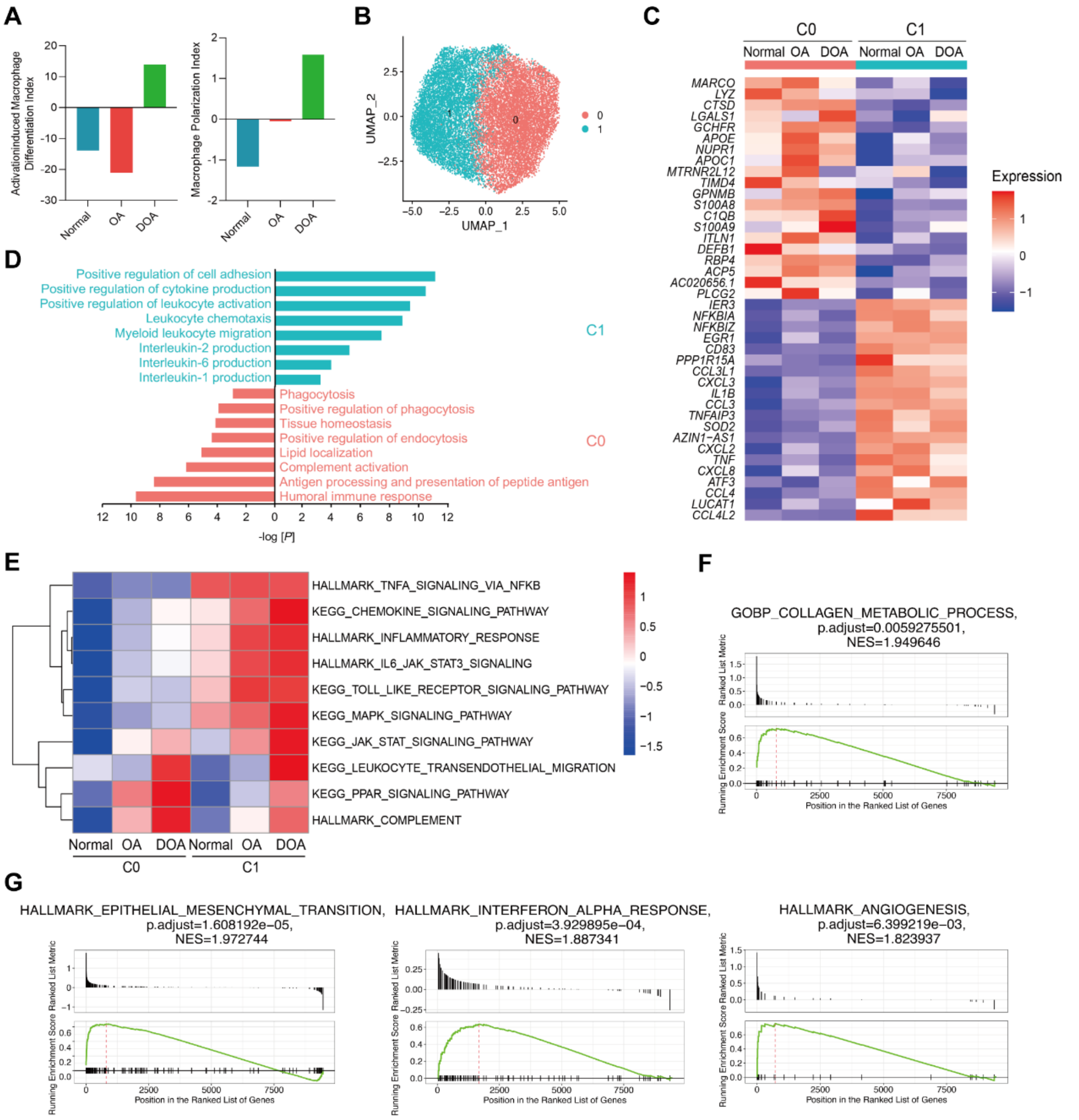

Figure S3. The enrichment of M1 macrophages in the synovium of DOA. (A-C) Macrophage Polarization Index (MPI) and Activation-induced Macrophage Differentiation Index (AMDI) defined by MacSpectrum in normal, OA, and DOA patients (A), and the UMAP visualization in panel (B) displays the C0 and C1 clusters with different colors. Panel (C) presents a heatmap showing the expression of specific markers associated with C0 and C1 macrophage subclusters across the patient groups. (D) Gene Ontology (GO) term analysis of C0 (a phagocytic macrophage) and C1 (an inflammatory macrophage) subclusters. (E) KEGG pathway enrichment analysis of macrophages in the synovium of normal, OA, and DOA patients. (F and G) Gene Set Enrichment Analysis (GSEA) enrichment plots for several representative signaling pathways that were upregulated in DOA compared to normal. The adjusted p-values and Normalized Enrichment Scores (NES) were shown in each pathway.

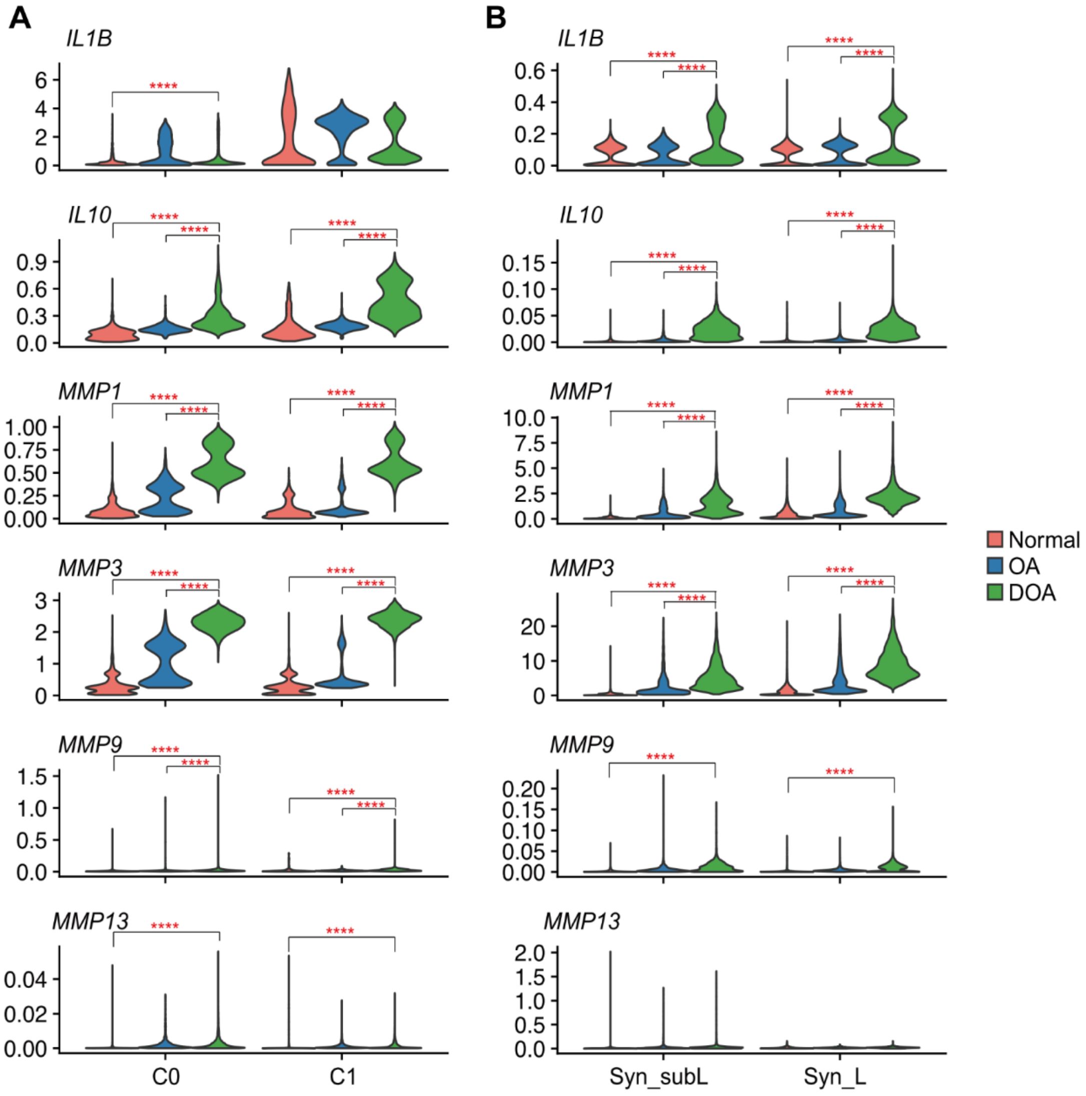

Figure S4. Cytokines and matrix metalloproteinases (MMPs) expression in synovial FLSs and macrophages across different patients. (A) Expression levels of the cytokines (*IL1B* and *IL10*) and the *MMPs* (*MMP1*, *MMP3*, *MMP9*, and *MMP13*) in C0 and C1 macrophages were analyzed. (B) Expression levels of the cytokines and the *MMPs* in the synovial FLSs were analyzed. ****$p < 0.0001$. Bimod test for (A and B).

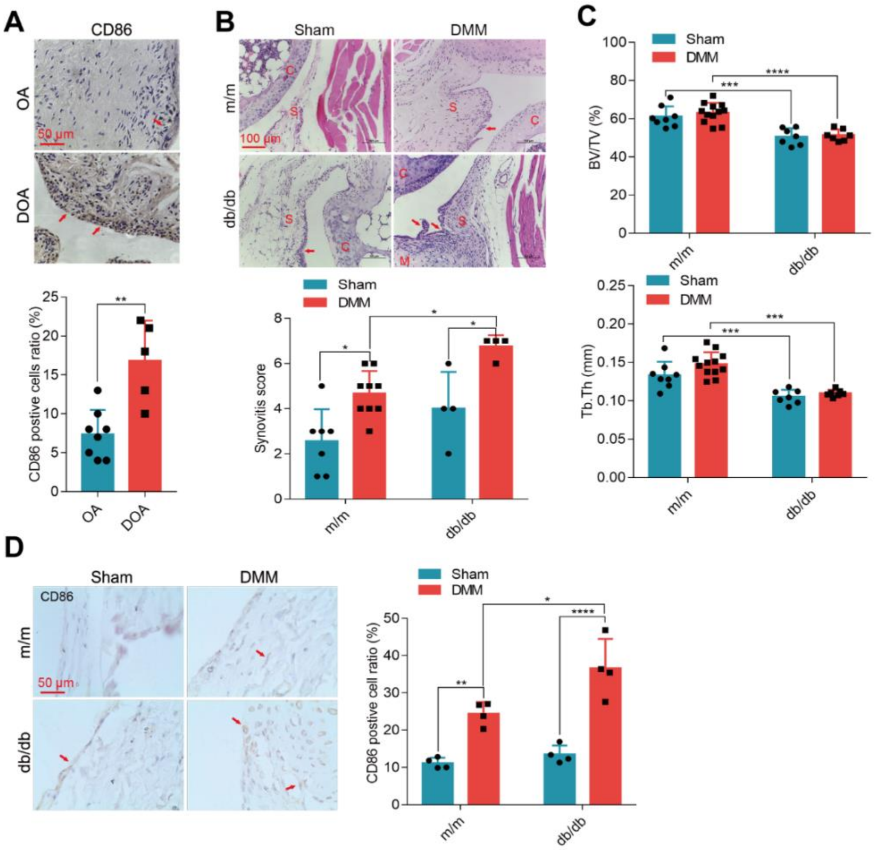

Figure S5. Diabetes mellitus exacerbates synovitis, OA-like change, and M1 macrophage activation. (A) Representative images of IHC staining and the quantification of $CD86^+$ cells in the synovium of OA and DOA patients (n ≥ 5). (B) HE staining and the quantification of synovitis scores of *m*/*m* (sham and DMM surgery) and *db*/*db* (sham and DMM surgery) mice (n ≥ 4). Scale bars, 100 μm. (C) Bone volume/tissue volume (BV/TV) and trabecular thickness (Tb. Th) were analyzed by micro-computed tomography (micro-CT) imaging (n ≥ 7). (D) Representative images of IHC staining and quantification of $CD86^+$ cells in the synovium of *m*/*m* (sham and DMM surgery) and *db*/*db* (sham and DMM surgery) mice (n = 4). Data are presented as mean ± SD, and dots represent individual mice. *$p < 0.05$, **$p < 0.01$, ***$p < 0.001$, and ****$p < 0.0001$. Student's t-test for (A); two-way ANOVA for (B-D).

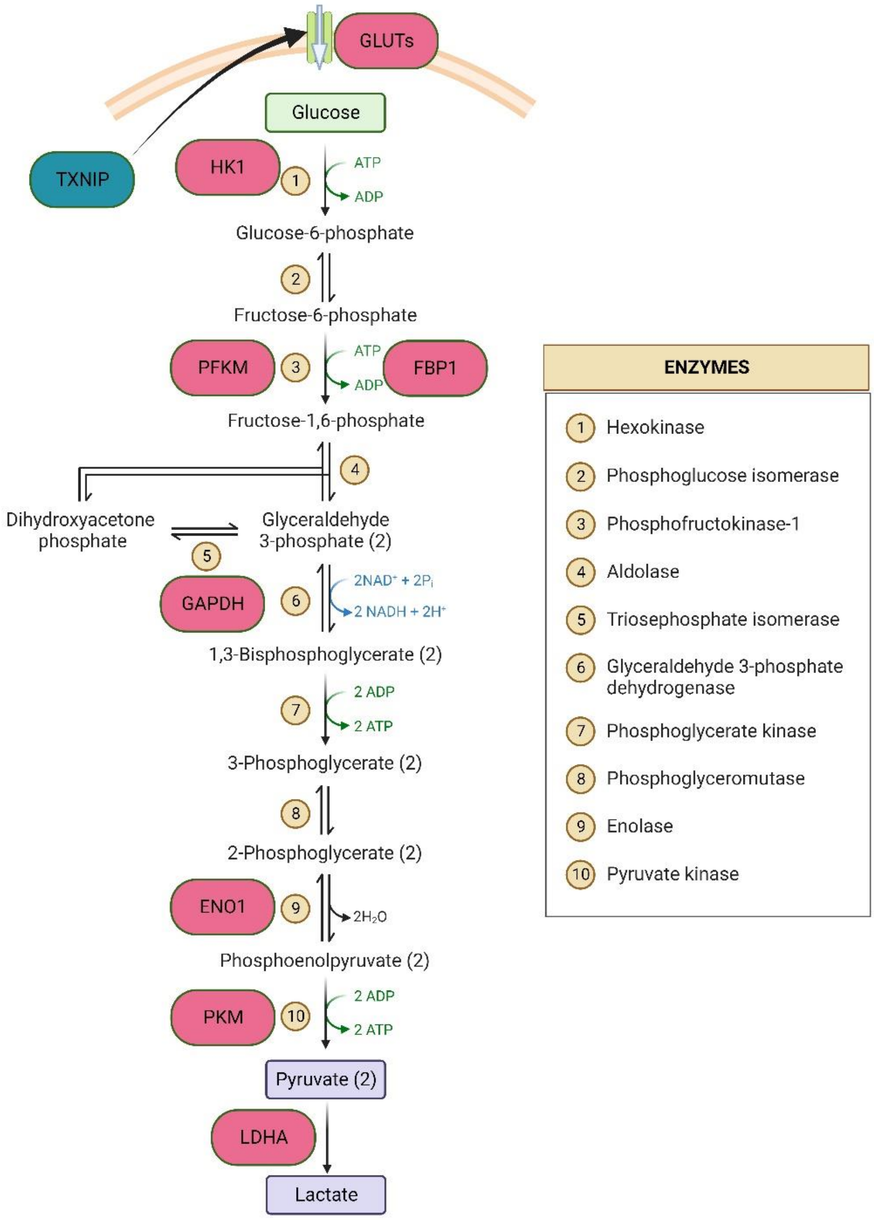

Figure S6. The altered glycolytic genes are annotated based on the scRNA-seq dataset. Several glycolytic genes, including thioredoxin-interacting protein (*TXNIP*), *SLC2A1 (GLUT1)*, *SLC2A5*, *HK1*, *PFKM (PFK1)*, *FBP1*, *GAPDH*, *ENO1*, *PKM*, and *LDHA* were defined and found to be altered. Red and blue colors represent increased or decreased gene expression, respectively.

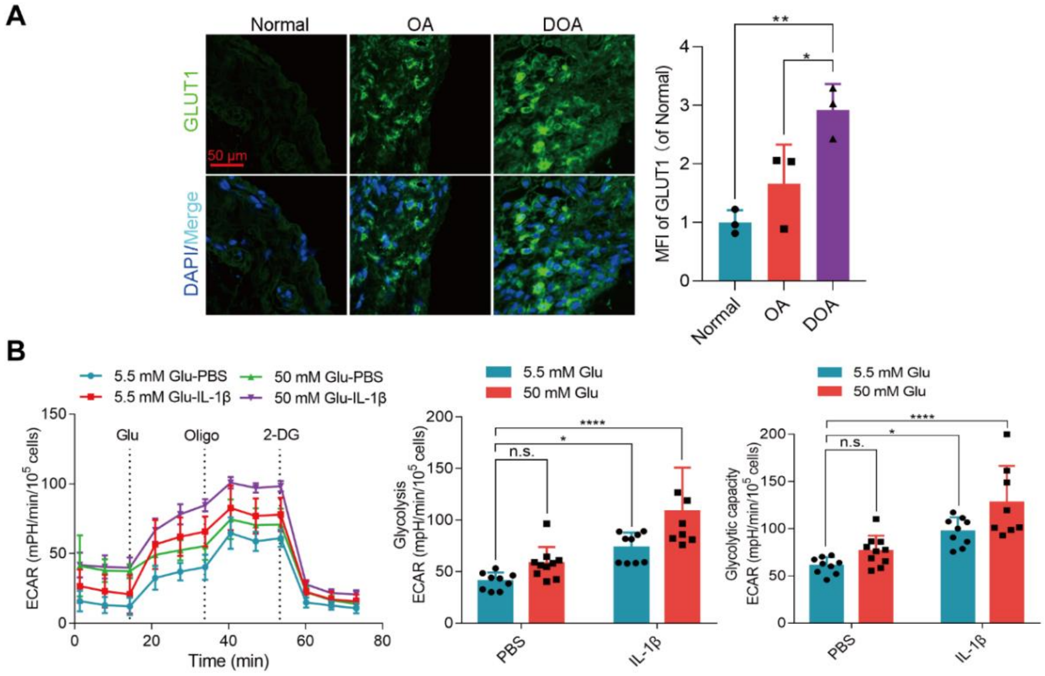

Figure S7. GLUT-1-dependent glycolysis is enhanced under stimulation by glucose and IL-1β. (A) Representative images of immunofluorescence (IF) staining of GLUT1 in the synovium of normal, OA, and DOA patients and the relative mean fluorescence intensity (MFI) of GLUT1 in the synovium were quantified. (B) Seahorse analysis assessed the extracellular acidification rate (ECAR), glycolysis, and glycolytic capacity in the FLSs treated with different glucose concentrations (5.5 mM or 50 mM) and stimulated with PBS or IL-1β for 24 h. Data are presented as mean ± SD. n.s.: not significant, *$p < 0.05$, **$p < 0.01$, **** $p < 0.0001$. One-way ANOVA for (A); two-way ANOVA for (B).

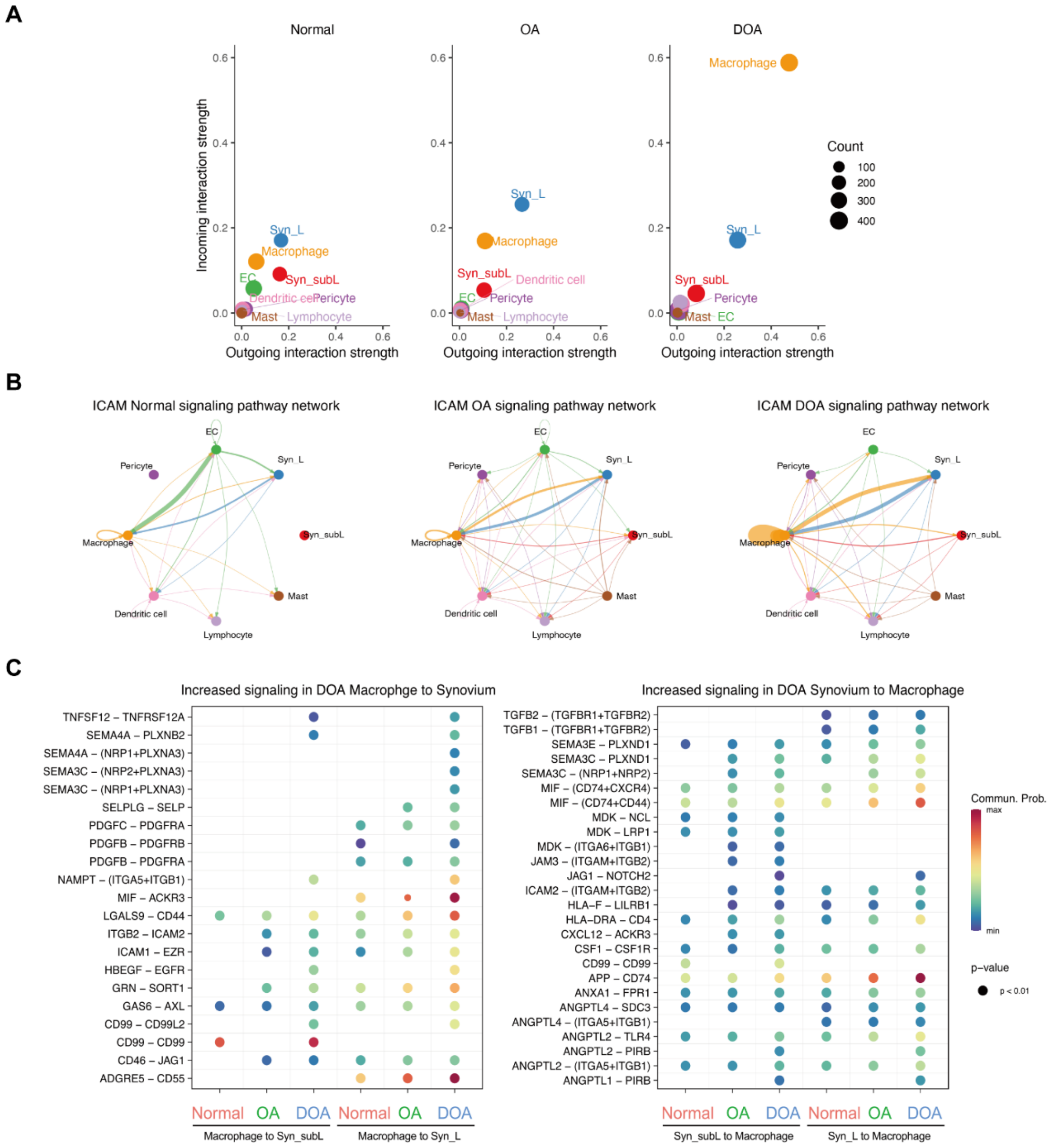

Figure S8. Cell-cell communication in the synovium of normal, OA, and DOA. (A) Scatter plots are presented to compare the outgoing and incoming interaction strengths in a 2D space among the three conditions (normal, OA, and DOA). (B) Circle plots are shown to display the inferred network of the ICAM signaling pathway in the synovium of normal, OA, and DOA. The edges represent communication probabilities, and the width is proportional to the inferred strength of communication between cell types. (C) Identification of increased signaling by comparing the communication probabilities mediated by ligand-receptor pairs between the synovium and macrophages.

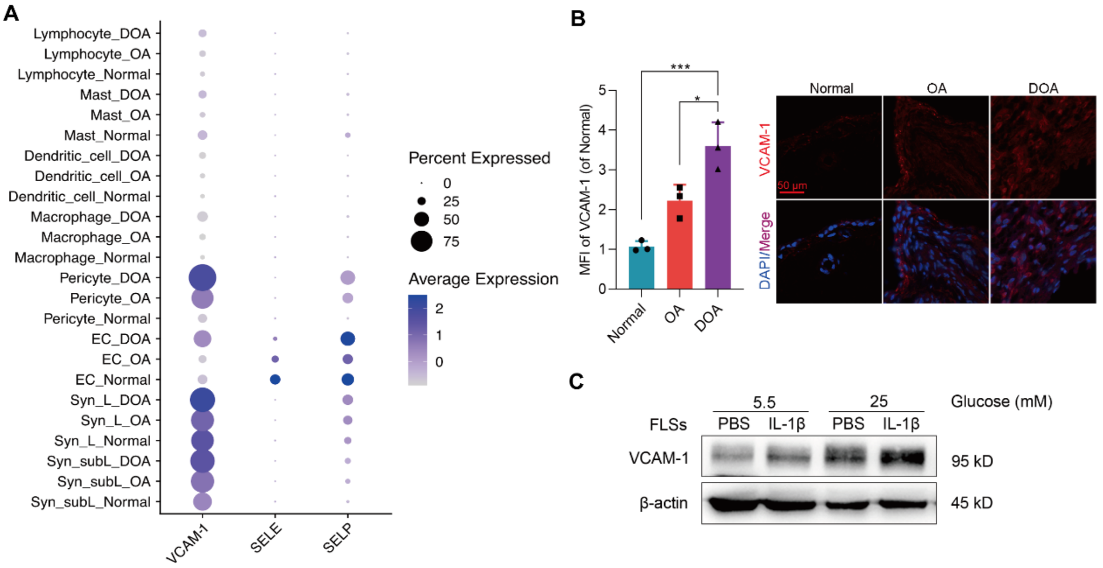

Figure S9. VCAM-1 expression in the FLSs under treatments and in patients with DOA. (A) The expression of VCAM-1 in different cell clusters was analyzed by scRNA-seq. (B) Representative fluorescent staining images of VCAM-1 were shown, and the relative MFI of VCAM-1 in the synovium was quantified (n = 3). (C) Immunoblotting of VCAM-1 expression in FLSs after treatment with different conditions, including PBS or IL-1β, and 5.5/25 mM glucose for 24 h. *p < 0.05, ***p < 0.001. One-way ANOVA for (B).

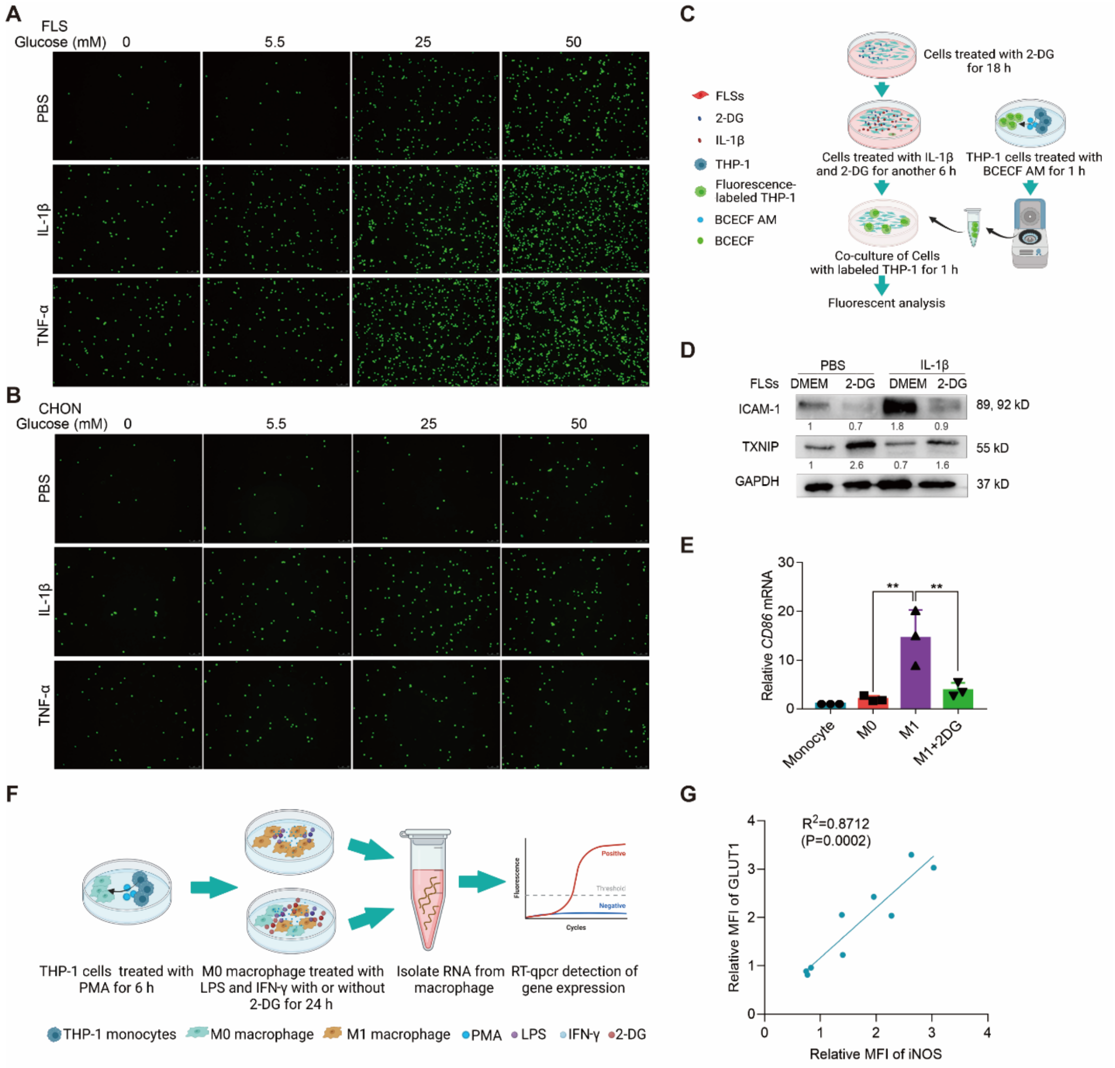

Figure S10. 2-DG inhibits high glucose- and inflammatory factors- induced monocyte/macrophage adhesion. (A and B) Representative images of monocyte adhesion are shown after FLSs (A) or CHON (B) were treated with different concentrations of glucose and inflammatory factors. (C) The schematic diagram illustrates the monocyte adhesion assay. FLSs were treated with PBS or IL-1β and either DMEM or 2-DG for 6 h. Fluorescently labeled THP-1 cells were then added to the FLSs for 1 h, then the adhesion of monocytes was assessed by imaging. (D) Immunoblotting of ICAM-1 and TXNIP protein expression in the FLSs after treatment with IL-1β and 2-DG for 24 h. (E and F) The schematic diagram illustrates the impact of 2-DG on macrophage polarization, specifically the mRNA expression of *CD86*, a marker of macrophage activation. (G) The correlation between the glycolytic level (represented by GLUT1 expression) and M1 macrophage polarization (iNOS) in the synovium of normal, OA, and DOA. Data are presented as mean ± SD. **p < 0.01. One-way ANOVA for (E).

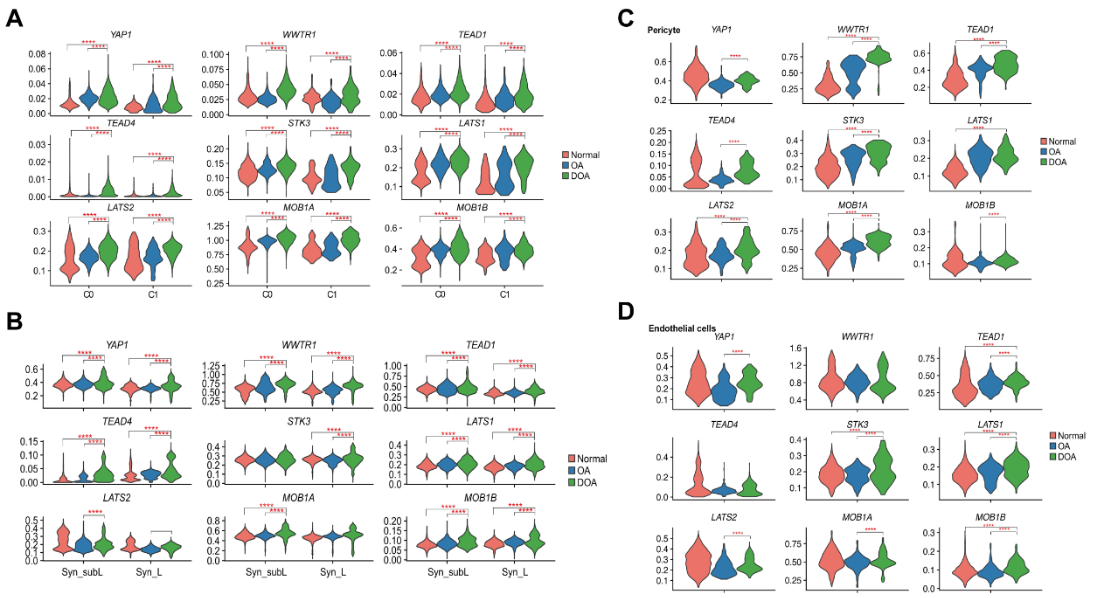

Figure S11. The Hippo-YAP1 signaling pathway is altered in the synovium of DOA. (A-D) Violin plots are used to visualize the expression levels of genes (*YAP1*, *WWTR1*, *TEAD1*, *TEAD4*, *STK3*, *LAST1*, *LAST2*, *MOB1A*, and *MOB1B*) associated with the Hippo-YAP1 pathway in different cell types, including macrophages, FLSs, pericytes, and endothelial cells (ECs), in the synovium of normal, OA, and DOA patients. ****$p < 0.0001$. bimod for (A, B, C and D).

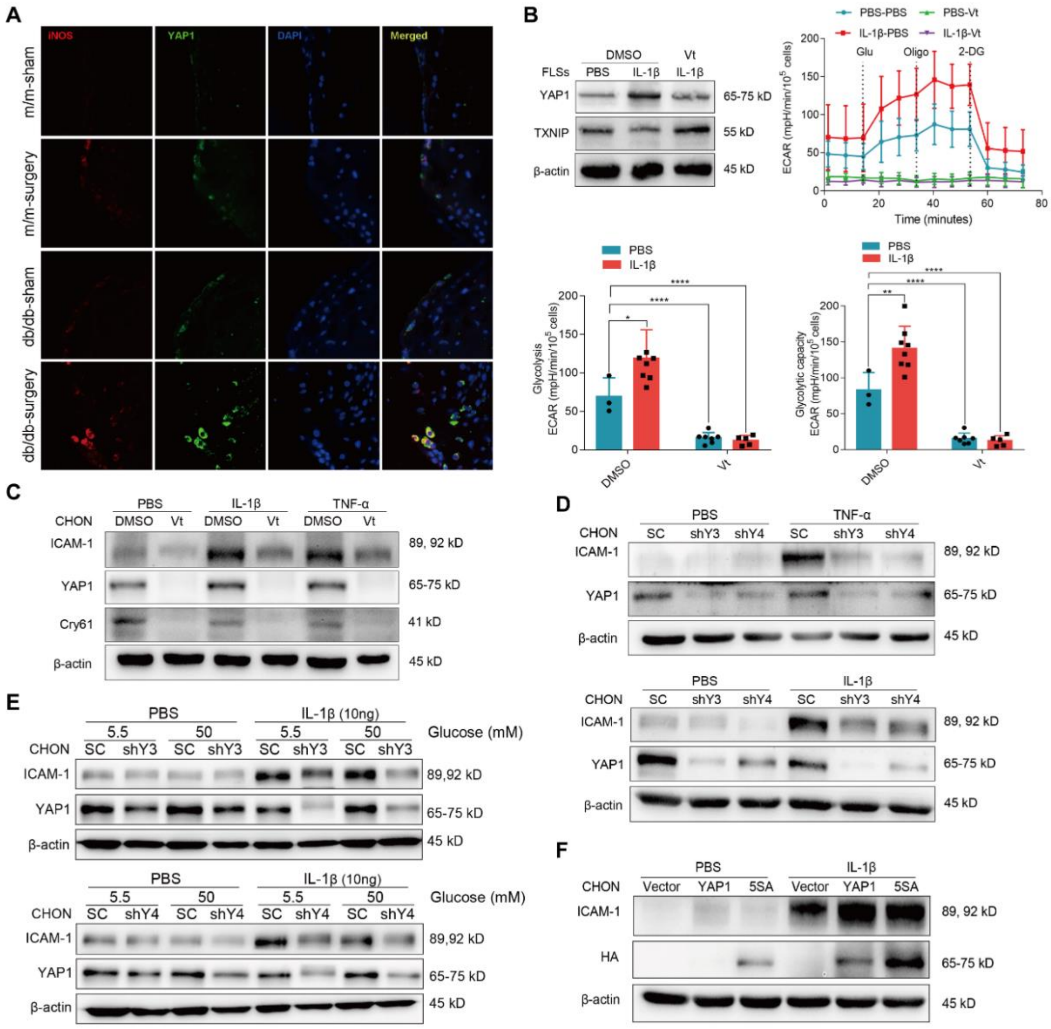

Figure S12. The role of YAP1 in regulating synovial glycolysis and the expression of adhesion molecules (e.g., ICAM-1). (A) Representative IF staining images showing the localization of iNOS (labeled in red) and YAP1 (labeled in green) in the synovium from knee joints of *m/m* (sham and DMM surgery) and *db/db* (sham and DMM surgery) mice. (B) Immunoblotting of YAP1 and TXNIP protein expression in the FLSs treated with IL-1β and Vt for 24 h. Seahorse analysis was applied to measure the extracellular acidification rate (ECAR), glycolysis, and glycolytic capacity in the FLSs treated with 50 mM glucose for 24 h, followed by the treatment with PBS or IL-1β and DMSO or Vt for an additional 24 h. (C) Immunoblotting analysis of ICAM-1, Cry61, and YAP1 protein expression in chondrocyte (CHON) treated with PBS, TNF-α, or IL-1β, and DMSO or Vt for 24 h. (D) Immunoblotting analysis of ICAM-1 and YAP1 protein expression in CHON after being transfected with SC (control), shY#3, or shY#4 for 48h, followed by treatment with PBS, TNF-α, or IL-1β for an additional 24 h. (E) Immunoblotting analysis of ICAM-1 and YAP1 protein expression in CHON treated with PBS or IL-1βand 5.5 or 50 mM glucose after being transiently transfected with SC or shY#3 for 48 h. (F) Immunoblotting analysis of ICAM-1 and YAP1 protein expression in CHON transiently transfected with vector, YAP1 (HA-YAP1), or 5SA (a constitutively active form of YAP1, HA-YAP1-5SA) for 48h, followed by the treatment with PBS or IL-1β for an additional 24 h. Data are presented as mean ± SD. *$p < 0.05$, **$p < 0.01$, ****$p < 0.0001$. Two-way ANOVA for (B).

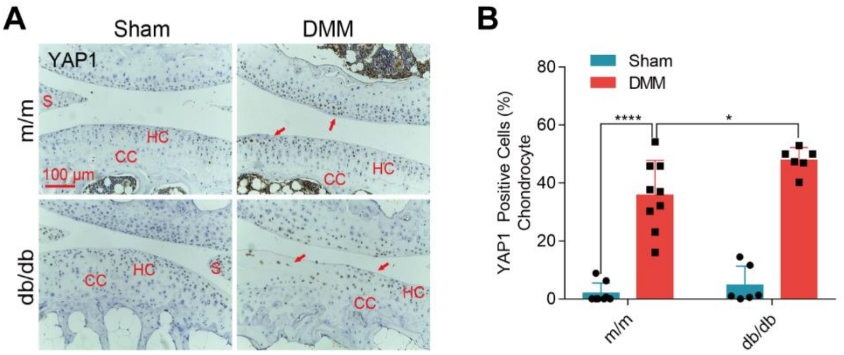

Figure S13. YAP1 is overexpressed in the synovium of the DMM surgery-induced mouse OA model. (A and B) Representative images of IHC staining for YAP1 and the quantification of YAP-stained positive cells in the knee joints of *m*/*m* (sham and DMM surgery) and *db*/*db* (sham and DMM surgery) mice (n ≥ 6). Data are presented as mean ± SD. *p < 0.05, and ****p < 0.0001. Two-way ANOVA for (B).

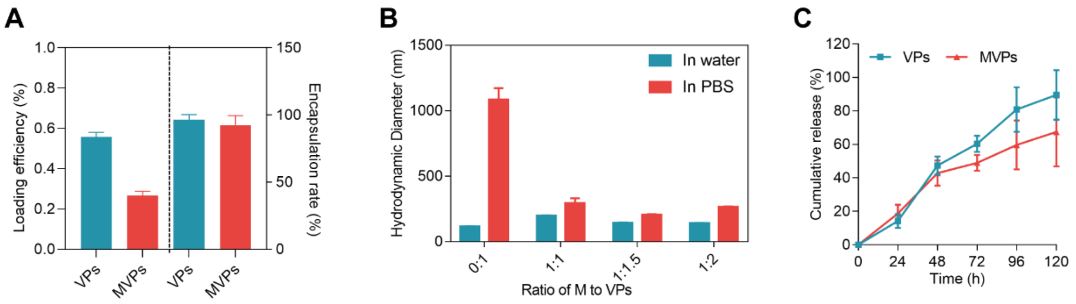

Figure S14. The characterization of MVPs. (A) Encapsulation efficiency (%) and loading efficiency (%) of VPs and MVPs. (B) Hydrodynamic diameters of MVPs were determined using different membrane-to-VPs protein weight ratios after sonication and after adjusting the solution to 1 X PBS. (C) The release kinetics of Vt from both VPs and MVPs were determined.

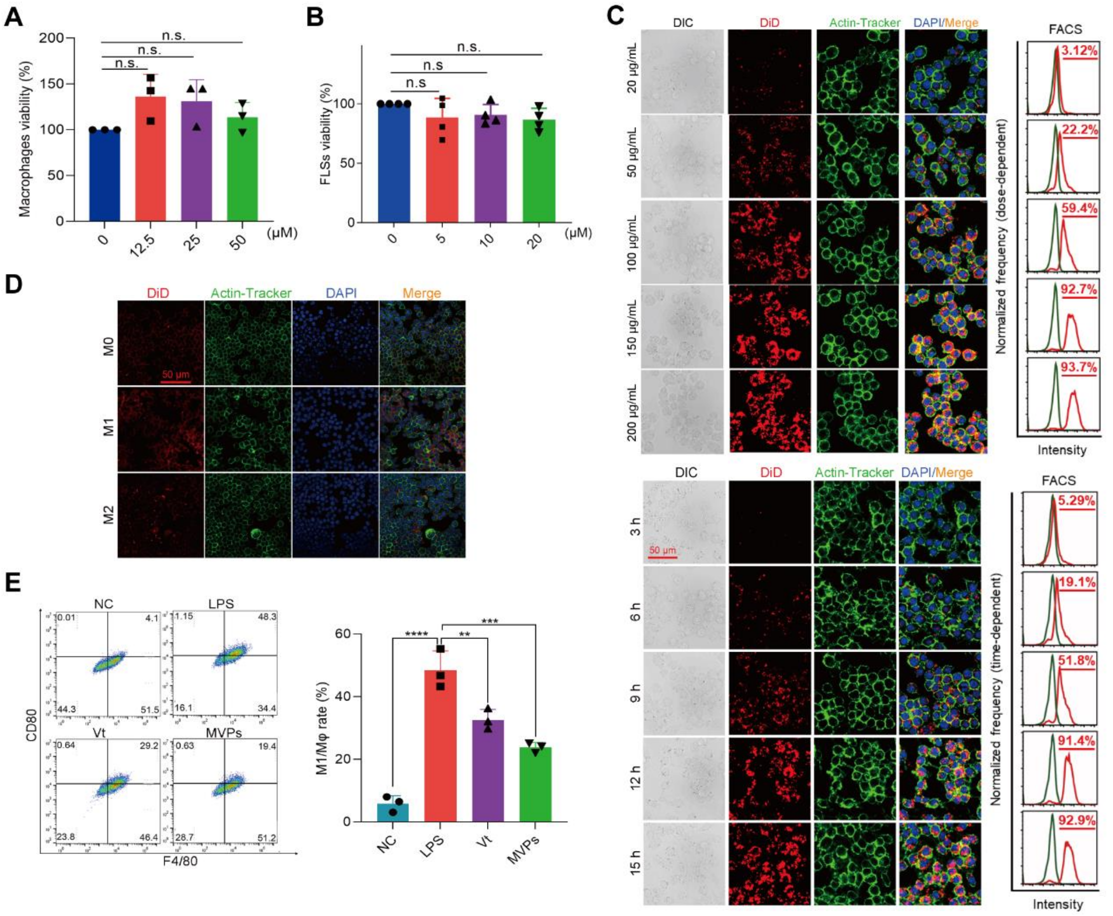

Figure S15. The effects of MVPs on cell viability and macrophage polarization *in vitro*. (A and B) Effect of MVPs on cell viability. (C) Dose- and time-dependent cellular uptake of different formulations determined by confocal laser scanning microscopy (CLSM) (left panel) and flow cytometry (FACS) (right panel). (D) RAW264.7 cells for induced for M1 and M2 differentiation, and then the cellular uptake of DiD-labeled MVPs was evaluated by co-staining with Actin-Tracker and DAPI. (E) RAW264.7 cells were treated with LPS in the presence of Vt pr MVPs for 24 h, and then the abundance of CD80 and F4/80 were measured by flow cytometry. Data are presented as mean ± SD. n.s.: not significant, $^{**}p < 0.01$, $^{***}p < 0.001$, and $^{****}p < 0.0001$. One-way ANOVA for (A, B, and E).

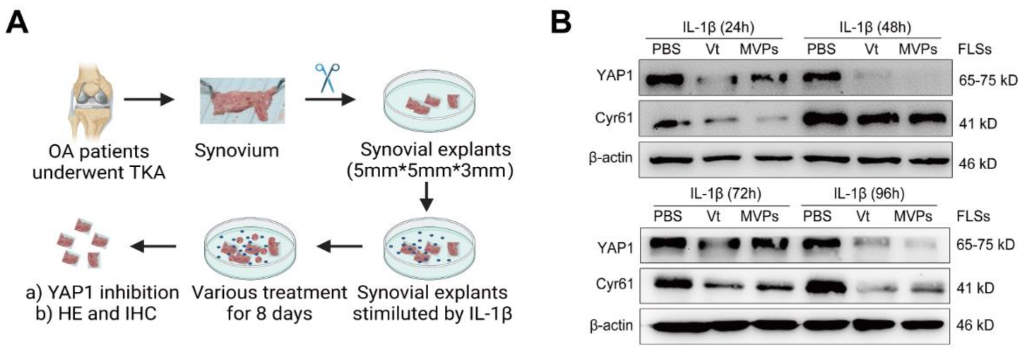

Figure S16. The effects of MVPs on synovial explants and the FLSs *in vitro*. (A) Schematic diagram of human synovial explants experiments. Briefly, synovium tissue was sectioned into 5 mm (length) × 5 mm (width) × 2 mm (depth) small explants with scissors and then cultured in a suitable medium for 2 days. After this initial culture period, the explants were treated with PBS or IL-1β, followed by the PBS, Vt, VPs, or MVPs for an additional 8 days. In the end, HE and IHC staining were performed. (B) Immunoblotting of YAP1 and its downstream protein (Cry61) expression in FLSs treated with PBS or IL-1β and in combination with PBS, Vt, or MVPs for varying periods (24, 48, 72, or 96 h).

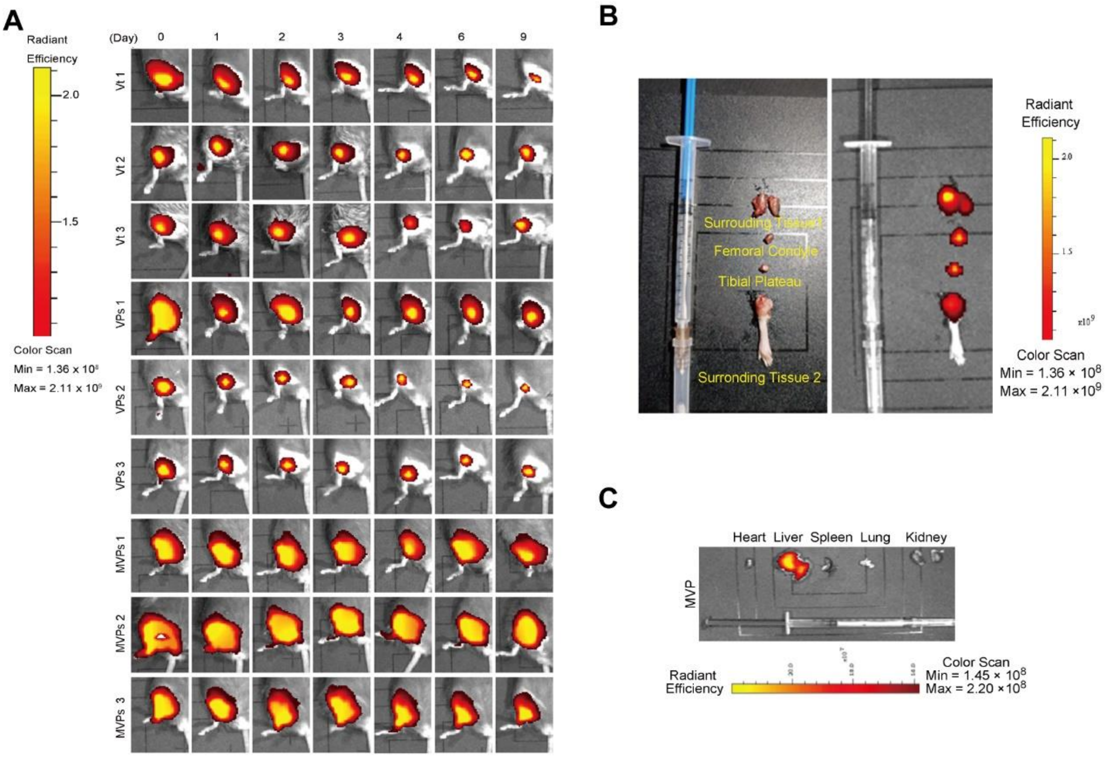

Figure S17. The biodistribution of MVPs and the cellular uptake of MVPs. (A) Images of mouse knee joints over 9 days after an intra-articular injection of DiR-labeled Vt, VPs, and MVPs. (B and C) Biodistribution of DiR-labeled MVPs in joint components (B) and internal organs (C) 24 h post single intra-articular injection.

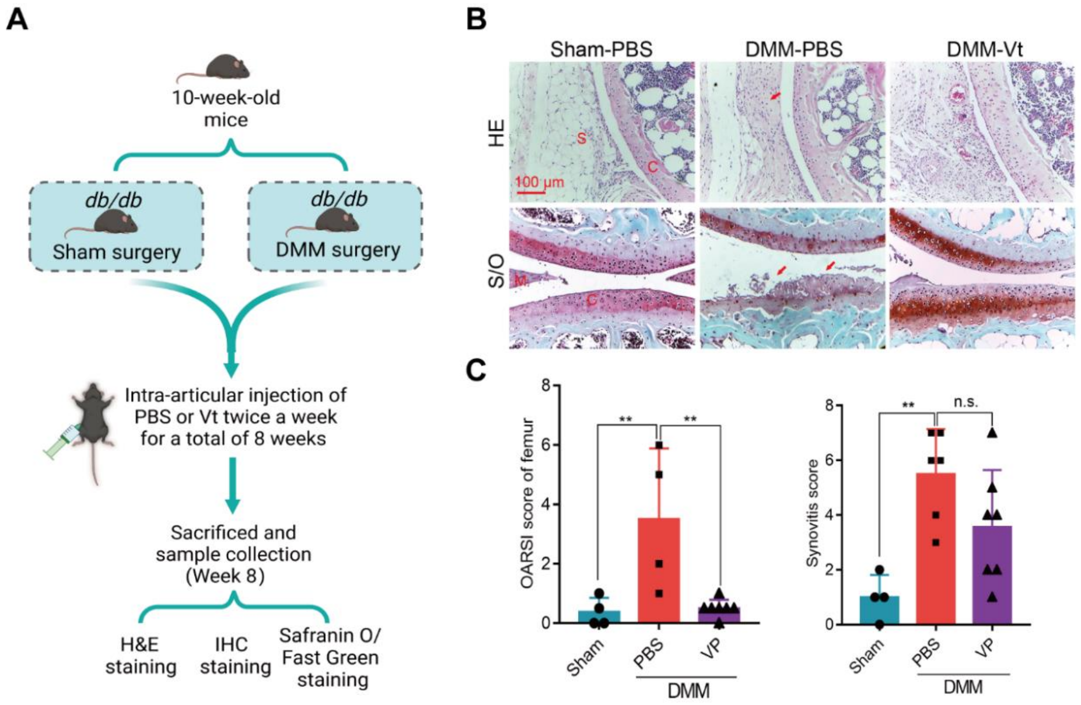

Figure S18. Vt attenuates joint destruction in the DOA mice model induced by DMM surgery. (A) Schematic diagram of the animal experiment design. Two groups of mice, *m/m* and *db/db*, at 10 weeks of age, underwent either sham surgery or DMM surgery to induce OA. Then, the mice were treated with intra-articular injections of PBS or VP immediately after the surgery and twice every week thereafter. Joints were harvested 8 weeks after surgery for further analysis. (B and C) HE and Safranin O/Fast Green staining of knee joints at 10 weeks after surgery (B), and the OA severity of knee joints was measured by the OARSI score and synovitis in C ($n \geq 4$). Data are presented as mean ± SD. n.s.: not significant, $^{**}p < 0.01$. One-way ANOVA for (C).

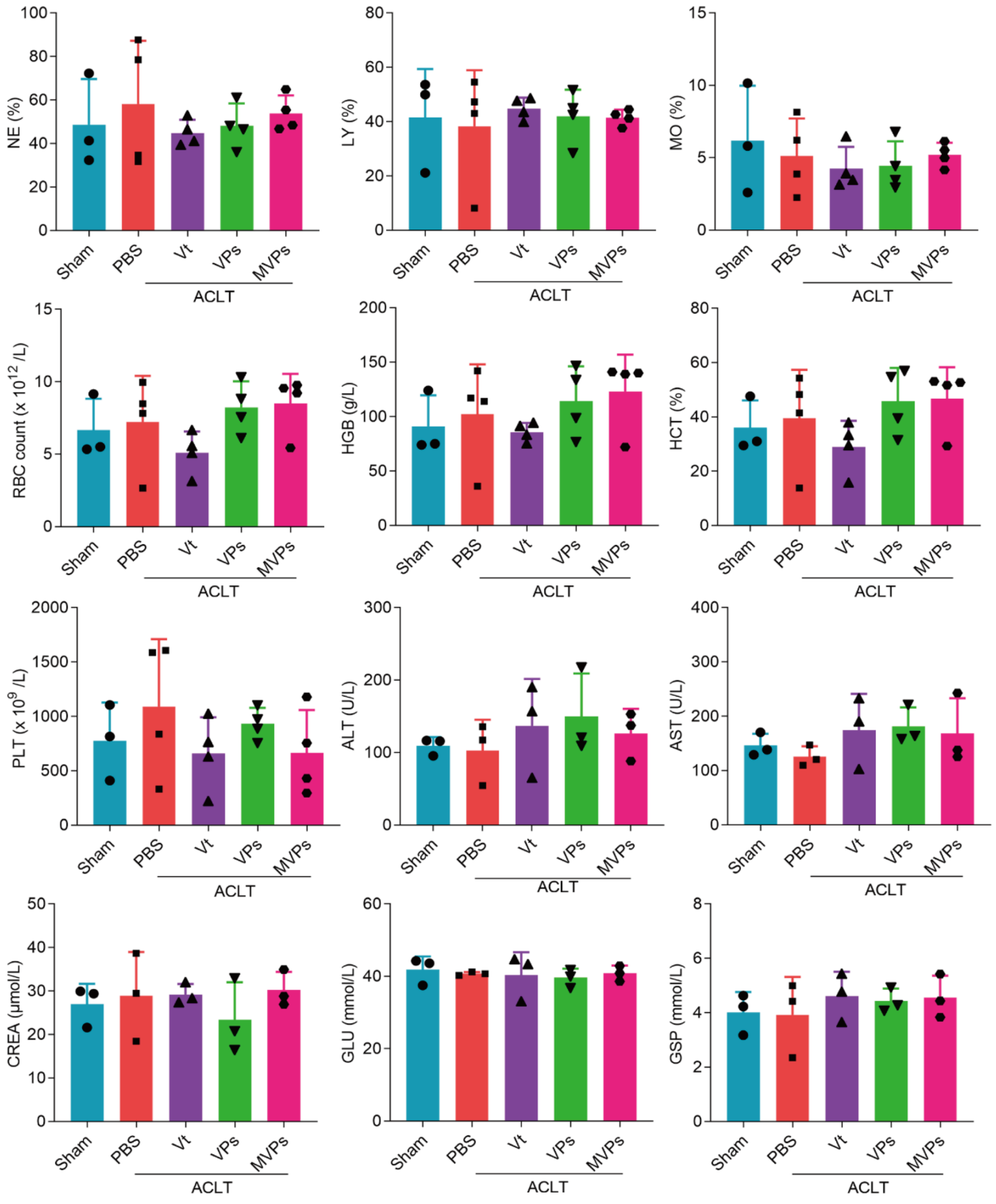

Figure S19. The effects of various treatments on the serum toxicology markers *in vivo*. Mice were subjected to 6-week serial injections, once every week, with different substances, including PBS, Vt, VPs, and MVPs. Hematologic parameters (NE (%), LY (%), MO (%), RBC counts, HGB, HCT (%), PLT counts, n ≥ 3), renal (CREA) and liver (ALT and AST) function, GLU and GSP were monitored (n = 3).

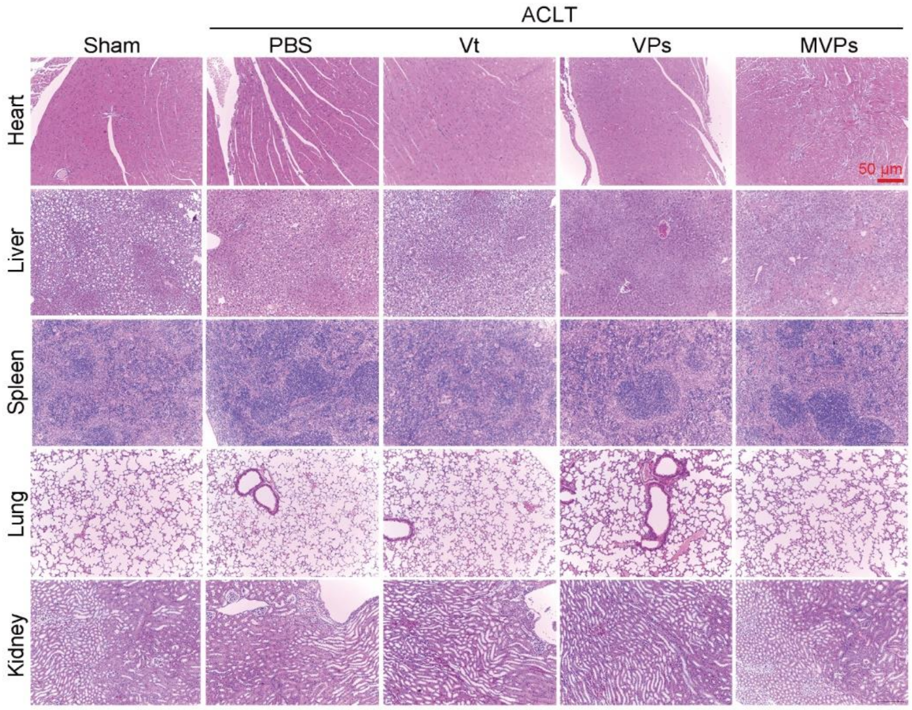

Figure S20. Histological evaluation of systemic toxicity *in vivo* after the indicated treatments. Representative images of HE staining of major organs (heart, liver, spleen, lung, kidney) after 6-week serial injections (once weekly) of PBS, Vt, VPs, and MVPs.

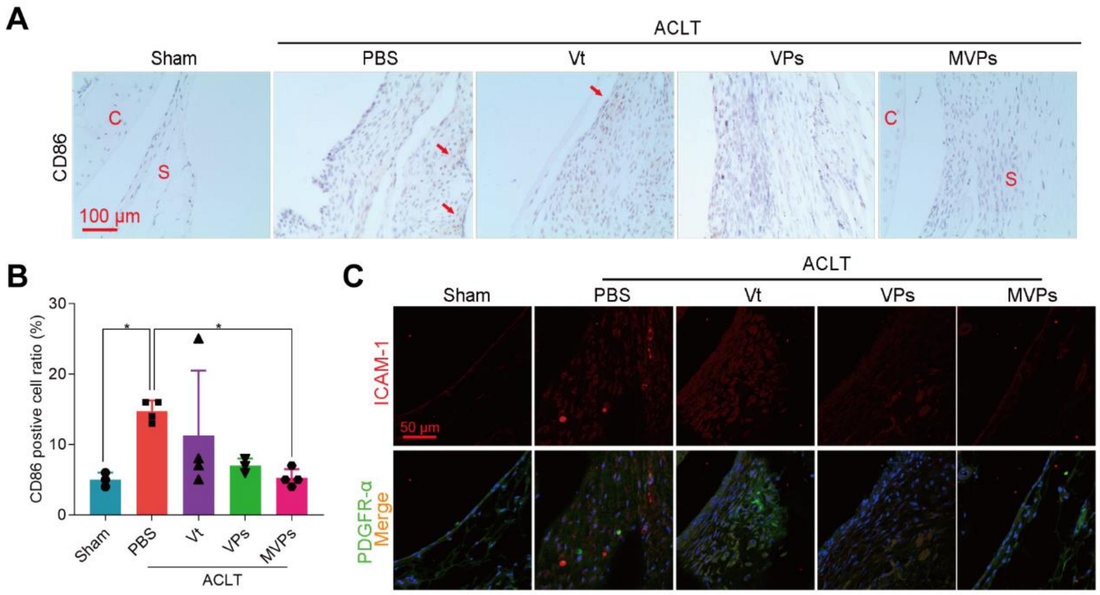

Figure S21. MVPs inhibit M1 macrophage polarization and ICAM-1 protein expression. (A and B) Representative images of IHC staining for CD86 (A) and quantification of the percentages of FLSs positive for CD86 staining (B) ($n \geq 3$). Representative fluorescent staining images for ICAM-1 (Intercellular Adhesion Molecule-1) along with PDGFR-α (Platelet-Derived Growth Factor Receptor Alpha). Data are presented as mean ± SD. $*p < 0.05$. One-way ANOVA for (B).

Table S3. Primer sequences for qPCR.

Gene	Genbank ID	Forward sequence	Reversed sequence
TXNIP	10628	GCCACACTTACCTTGCCAAT	TGATCTTCTGAACCCGAAGG
ICAM-1	3383	CGACTGGACGAGAGGGATTG	GGAGAGCACATTCACGGTC
CD86	942	CGTTTCCATCAGCTTGTCTGT	CGTCTTGTCAGTTTCCAGAATAC
CCL2	6347	GATCTCAGTGCAGAGGCTCG	TTTGCTTGTCCAGGTGGTCC
MMP13	4322	GCGGGAATCCTGAAGGAGAAT	TTTGCCAGTCACCTCTAAGCC
MMP1	4312	GGTGATGAAGCAGCCCAGAT	GATGTGTTTGCTCCCAGCGA

Table S4. Key resources

REAGENT or RESOURCE	SOURCE	IDENTIFIER
Antibodies		
Anti-rabbit ICAM-1	Abcam	Cat# ab109361
Anti-rabbit YAP1	Cell Signaling Technology	Cat# 14074
Anti-rabbit P-YAP1	Cell Signaling Technology	Cat# 13008
Anti-mouse TXNIP	Abcam	Cat# ab210826
Anti-rabbit TXNIP	Abcam	Cat# ab188865
Anti-rabbit Cyr61	Cell Signaling Technology	Cat# 14479
Anti-rabbit MMP13	Abcam	Cat# ab39012
Anti-mouse PDGFR-α	Santa Cruz	Cat# sc-398206
Anti-rabbit iNOS	Abcam	Cat# ab3523
Anti-rabbit iNOS	Servicebio	Cat# GB11119
Anti-rabbit VCAM-1	Cell Signaling Technology	Cat# 13662
Anti-rabbit CD86	Abcam	Cat ab239075
FITC anti-mouse CD80	Biolegend	Cat# 104705
PE anti-mouse F4/80	Biolegend	Cat# 123110
Anti-rabbit HA	Cell Signaling Technology	Cat# 2367
β-actin	Cell Signaling Technology	Cat# 3700
Anti-rabbit IgG H&L (HRP Conjugated)	Cell Signaling Technology	Cat# 7074
Anti-mouse IgG H&L (HRP Conjugated)	Cell Signaling Technology	Cat# 7076
Anti-rabbit IgG (H+L) (Alexa Fluor® 555 Conjugate)	Cell Signaling Technology	Cat# 4413
Anti-rabbit IgG (H+L) (Alexa Fluor® 488 Conjugate)	Cell Signaling Technology	Cat# 4412
Anti-mouse IgG (H+L) (Alexa Fluor® 488 Conjugate)	Cell Signaling Technology	Cat# 4408
Anti-mouse IgG (H+L) (Alexa Fluor® 555 Conjugate)	Cell Signaling Technology	Cat# 4409
Biological samples		
Fibroblast-like synoviocytes (FLSs)	Patients in this study	N/A
Chondrocytes (CHON)	Patients in this study	N/A
Synovium	Patients in this study	N/A
Chemicals, peptides, and recombinant proteins		
Verteporfin	Selleck Chemicals	S1786
IL-β	PeproTech	200-01B-10
Poly (lactic-co-glycolic acid)	Aladdin	P133297
BCECF	Beyotime	S1006
DiR	YEASEN	CD4666
DiD	YEASEN	CD4665
DAPI	Cell Signaling Technology	4083S
SYBR Green	Applied Biosystems	4385617
TRIzol reagent	Takara	9109

Fetal bovine serum (FBS)	Gibco	10099141C
Critical commercial assays		
High-Capacity cDNA Reverse Transcription Kit	Invitrogen	4368814
Membrane Extraction Sample Kit	Invent	SM-005-50T
Saffron-O and Fast Green Stain Kit	Servicebio	G1053
Tsaplus Fluorescence Triple Staining Kit	Servicebio	G1236
H&E staining kit	Leagene	CM0686
Deposited data		
Single-cell RNA seq data	This paper	NGDC: PRJCA015031
Experimental models: Cell lines		
RAW 264.7	ATCC	ATCC TIB-71
THP-1	ATCC	ATCC TIB-202
Experimental models: Organisms/strains		
db/*db* diabetic mice	GemPharmatech Co., Ltd	T00240
m/*m* non-diabetic mice	GemPharmatech Co., Ltd	N000214
